# Abstracts of the 2013 AAPM Spring Clinical Meeting

**DOI:** 10.1120/jacmp.v14i3.4458

**Published:** 2013-05-06

**SATURDAY, MARCH 16**

**BEST POSTER COMPETITION EXHIBIT HALL**

**PO‐BPC‐Exhibit Hall‐01**

## Evaluation of Dose Reduction with Philips iDose Reconstrutcion in Relations to image Quality in a Phantom Study

T Hameed, Y Liang,^*^ J Sheng, J Rydberg

*Indiana University, Indianapolis, IN*

### Purpose

To investigate the use of a commercially designed hybrid iterative reconstruction technique iDose (by Philips Medical Systems) for CT radiation dose reduction and its effect on image quality.

### Methods

A catphan504 phantom was scanned using a 64‐slice CT (Ingenuity, Philips Medical Systems) with two different tube voltages (100 kVp, and 120 kVp) at three different doses, a reference dose (CTDIvol) of 42 mGy and two reduced doses of about 50% and 75% of the reference. Images were reconstructed with standard filtered‐back‐projection (FBP) and with iDose algorithms. Six different iDose levels were employed. Quantitative evaluation of spatial resolution, image noise, noise power spectrum (NPS), and low‐contrast detectability were carried out.

### Results

For any given dose level, there was a static noise reduction with increased iDose level over the FBP. To match the standard noise resulted from the FBP at the reference dose, a minimum iDose level of 4 and 6 was required for 50% and 75% dose reduction. respectively. NPS showed moderate shift towards the lower frequency as iDose level increased. The NPS shift was consistent with the observed subtle change of noise texture. This shift also correlated with the change in low‐contrast detectability among images with the same noise level: the higher the iDose level that was used in image reconstruction, the lower the low‐contrast detectablity.

### Conclusion

The iDose algorithm clearly demonstrates effectiveness in noise suppression over the FBP. The low‐contrast detectability depends on noise. but also on NPS, which is shifted by iDose algorithm. At very low‐dose levels, greater iDose levels would be needed to reduce the image noise, but may not improve the low‐contrast detectabilty. These findings indicate that for any given specific clinical task, the lowest dose limit in combination with an optimal iDose level may be established by considering both noise and low‐contrast detectability desired.

**PO‐BPC‐Exhibit Hall‐02**

## Quantifying Similarity of Strut‐adjusted Volume implant (SAVi) Applicator in Accelerated Partial Breast irradiation (APBi) HDR Bachytherapy

K Li^1,2*^ J Newton^1,2^

*John R. Marsh Cancer Center,^1^ Hagerstown, MD, Associates in Medical Physics,^2^ Lanham, MD*

### Purpose

In order to provide guidelines to determine the appropriateness for HDR Brachytherapy with strut‐adjusted volume implant (SAVI) applicator, the different magnitudes of distortion, rotation and displacement were quantitatively analyzed by a distance comparison method based on markers built in SAVI applicator.

### Methods

A SAVI 8‐1 applicator was used for this study. After a CT scan of the applicator, a set of digitally reconstructed radiographs (DRR) for orthogonal fields were generated as a reference and used to check the consistency of the applicator during treatment course. Then, the SAVI applicator was set up on the table using three different scenarios, which are parallel rotation, central axis rotation, and diameter variation of applicator. These three scenarios included all the possibilities of the patient alignment and displacement. The sum of the marker‐to‐marker distance was used to evaluate the similarity level of the SAVI applicator due to different setup variations.

### Results

In this study, given the full expansion of SAVI applicator, for parallel rotation, using anterior–posterior (AP) imaging, the average distance sum for three markers was 9.56 cm, and STDV was 0.02 cm. For axial rotation, using AP imaging, the average distance sum was 9.7 cm, and STDV was 0.3 cm; and using Lateral imaging, the average distance sum was 7.4 cm and STDV was 1.5 cm. For AP imaging with a 45° alignment of the applicator, as the diameter went from 3.5 cm to 1.5 cm, the distance sum was not highly sensitive; however, laterally the distance sum changed from 9.1 cm to 6.4 cm.

### Conclusion

Clinically, it is better to check both of the marker distance sum and furthest strut distance, as well as skin mark, to determine the appropriateness for APBI HDR treatment. Further outcome studies should develop a safe treatment margin based on translation and rotational matrix as with given marker coordinates.

**PO‐BPC‐Exhibit Hall‐03**

## Implementation of High‐Dose‐Rate Grid Therapy Using a 6 MV Flattening‐Filter‐Free Photon Beam

F Lerma^*^ K Dou, M Jacobs, B Li, B Lee, G Casados, S Arthur, B Lanahan

*Mercy Medical Center and RadAmerica LLC–MedStar Health, Baltimore, MD*

### Purpose

Implement high‐dose rate grid therapy using a flattening‐filter‐free 6 MV beam to deliver single‐fraction 12 – 20 Gy treatments to debulk tumors that do not respond to conventional fractionation schemes.

### Methods

Grid therapy involving a 6 MV flattening‐filter‐free (FFF) photon beam, shaped with 5 mm leaves of a Millenium 120 multileaf collimator (MLC) into 1 cm x 1 cm beamlets spaced 1 cm apart, giving an effective blocking of 75% is planned on a Varian Medical Systems’ Eclipse RTPS V10.0. The resulting plans are delivered on a TrueBeam linear accelerator at 1400 MU per minute. Quality Assurance is conducted using a comparison of computed dose planes in a flat, virtual water phantom against diode array measurements at 5 cm depth, 100 SAD in a MapCheck 2 detector, by Sun Nuclear Corporation. Patient treatments are comprised of one or more beams, 5 to 6 cm wide, and 10 to 16 cm long, where abutting fields are achieved by shifting the MLC pattern and the x‐jaws on the linear accelerator. Image guidance (IGRT) maximizes treatment setup accuracy and speed. Total treatment time, beam‐on time, and dose rate are minimized by the combination of image guidance, FFF beam operation, and field abutment using jaw and MLC movements.

### Results

1500 to 4000 MU deliver 12 to 20 Gy using 1 – 3 abutting grid therapy beams, in 5 to 12 minutes, in contrast to extended treatment times of more than 30 minutes at 600 MU/min without IGRT or abutting field planning. Gamma analyses show 98% pass rate at 2.2 mm and 2.2% thresholds.

### Conclusion

MLC‐based, high‐dose‐rate FFF 6 MV beam grid therapy under image guidance is planned, verified, and delivered, thus demonstrating a sharp reduction in treatment times, and targeting accuracy in MLC‐based grid therapy methods.

**PO‐BPC‐Exhibit Hall‐04**

## Verification and Dosimetric Impact of Acuros XB Algorithm for Stereotactic Body Radiation Therapy (SBRT) and RapidArc Planning for non‐Small‐Cell lung Cancer (nSClC) Patients

S Rana,^1*^ K Rogers^2^

*ProCure Proton Therapy Center,^1^ Oklahoma City, OK, Arizona Center for Cancer Care,^2^ Peoria, AZ*

### Purpose

The experimental verification of Acuros XB (AXB) algorithm was conducted in a heterogeneous slab phantom, and compared to Anisotropic Analytical Algorithm (AAA). The dosimetric impact of AXB for stereotactic body radiation therapy (SBRT) and RapidArc planning for 16 non‐small‐cell lung cancer (NSCLC) patients was assessed.

### Methods

The calculated central axis percentage depth doses (PDD) in a phantom for an open field size $\left(3\times 3\;{\text{cm}}^{2}\right)$ were compared against PDD measured by an ionization chamber. For 16 NSCLC patients, dose‐volume parameters from treatment plans calculated by AXB and AAA were compared using identical jaw settings, leaf positions, and monitor units (MUs).

### Results

The results from the phantom study showed that AXB was more accurate than AAA; however, dose underestimation by AXB (up to −3.9%) and AAA (up to −13.5%) was observed. For a planning target volume (PTV) in NSCLC patients, in comparison to AAA, the AXB predicted lower mean and minimum doses by average 0.3% and 4.3%, respectively, but a higher maximum dose by average 2.3%. The averaged maximum doses to heart and spinal cord predicted by AXB were lower by 1.3% and 2.6%, respectively; whereas doses to lungs predicted by AXB were higher by up to 0.5% compared to AAA. The percentage of ipsilateral lung volume receiving at least 20 and 5 Gy were higher in AXB plans than in AAA plans by average 1.1% and 2.8% respectively. The AXB plans produced higher target heterogeneity by average 4.5% and lower plan conformity by average 5.8%, compared to AAA plans. Using AXB, the PTV coverage was reduced by an average of 8.2% than when using AAA.

### Conclusion

AXB is more accurate to use for dose calculations in SBRT lung plans created with a RapidArc technique; however, one should also note reduced PTV coverage due to dose recalculation from AAA to AXB.

**PO‐BPC‐Exhibit Hall‐05**

## Use of Blade Sequences in lumbar Spine MR imaging for eliminating Motion, Pulsatile Flow, and Cross‐talk Artifacts

P Mavroidis^1^ E Lavdas^2^ S Kostopoulos^2^ D Glotsos^2^ V Roka^3^ A Koutsiaris^4^ G Batsikas^5^ G Sakkas^6^ A Tsagkalis^5^ I Notaras^7^ S Stathakis^8^ N Papanikolaou^1*^ K Vassiou^7^

University of Texas Health Science Center (UTHSCSA),^1^ San Antonio, TX, USA and Karolinska Institutet and Stockholm University, Sweden, Techological Education Institute of Athens,^2^ Athens, Greece, Health Center of Farkadona,^3^ Trikala, Greece, Technological Educational Institute of Larissa,^4^ Larissa, Greece, IASO Thessalias Hospital,^5^ Larissa, Greece, Center for Research and Technology,^6^ Thessaly, Trikala, Greece, University of Thessaly,^7^ Larissa, Greece, Cancer Therapy and Research Center^8^

### Purpose

The purpose of this study is to evaluate the ability of T2 Turbo Spin Echo (TSE) AXIAL and SAGITAL BLADE sequences in reducing or even eliminating motion, pulsatile flow, and cross‐talk artifacts in lumbar spine MRI examinations.

### Methods

Forty‐four patients, who had routinely undergone a lumbar spine examination, participated in the study. The following pairs of sequences with and without BLADE were compared: a) T2 TSE Sagittal (SAG) in thirty‐two cases, and b) T2 TSE Axial (AX) also in thirty‐two cases. Both qualitative and quantitative analyses were performed based on the signal‐to‐noise ratio (SNR), and contrast‐to‐noise ratio (CNR), measures of different normal anatomical structures. The qualitative analysis was performed by experienced radiologists. Also, the presence of image motion, pulsatile flow, and cross‐talk artifacts was evaluated.

### Results

Based on the results of the SNR and CRN for the different sequences and anatomical structures, the BLADE sequences were found to be remarkably superior to the conventional ones in all the cases. The same findings were also observed in the qualitative analysis. The BLADE sequences eliminated the motion artifacts in all the cases. In our results, it was found that in the examined sequences (Sagittal and Axial) the differences between the BLADE and conventional sequences regarding the elimination of motion, pulsatile flow, and cross‐talk artifacts were statistically significant. In all the comparisons, the T2 TSE BLADE sequences were significantly superior to the corresponding conventional sequences regarding the classification of their image quality.

### Conclusions

This technique appears to be capable of potentially eliminating motion, pulsatile flow, and crosstalk artifacts in lumbar spine MR images and producing high‐quality images in collaborative and noncollaborative patients.

**PO‐BPC‐Exhibit Hall‐06**

## Is Similarity a Useful Tool in Assessing Quality of Prostate Brachytherapy Seed implants?

D Todor^*^ M Hagan, M Anscher

*Virginia Commonwealth University, Richmond, VA*

### Purpose

To identify whether geometrical similarity between a preplan and postimplant plan is necessary and/or sufficient for a good quality implant?

### Methods

A unique cohort of 94 prostate seed implant patients was analyzed. For each patient, an ultrasound (US) preimplant and two CT postimplant (day 0 and day 30) studies were available. Measures for similarity were created and computed using feature vectors based on two classes of moments: first, invariant to rotation and translation, the second polar‐radius moments invariant to rotation, translation, and scaling. Both similarity measures were calibrated using controlled perturbations (random and systematic) of seed positions and contours in different size implants, thus producing meaningful numerical threshold values used in the clinical analysis.

### Results

When similarity is based on moments invariant to translation and rotation only, both seeds and contours exhibit a remarkable lack of similarity when pre‐ and postimplant plans are compared, with contours faring slightly better than seeds. When scaling invariance is added, resemblance between both implants and contours improved dramatically. When comparing postimplant plans at day 0 and day 30, apart from minor seed migration, the only change in the geometry of the implant should be due to resolution of prostate swelling and should be well describe by scaling, and this is clearly shown by the 83% (78 patients out of 94) similarity. Interestingly, there is no correlation between seed similarity and contours similarity, nor does D90 or D90 ratios correlate with seed or contours similarity in any of the comparisons.

### Conclusion

In the setting of preplanned prostate seed implants using preloaded needles as delivery method, based on our data we conclude that similarity between pre‐ and postimplant plan does not correlate with either D90 or analogous similarity metrics for prostate contours, thus questioning the utility of creating ‘optimal’ preplans.

**PO‐BPC‐Exhibit Hall‐07**

## Experimental evaluation of RF Shield at 1.5 and 3.0 Tesla MRi

C Favazza,^*^ K Gorny, J Felmlee

*Mayo Clinic, Rochester, MN*

### Purpose

To evaluate radiofrequency (RF) shield (Accusorb, Axonics, Inc. USA) for prospective use during 1.5T and 3.0T MRI examinations of patients with implanted metallic devices.

### Methods

To assess attenuation of RF field, a standard 60×132 cm blanket (Accusorb, Axonics, Inc., USA) was wrapped around an acrylic tube and positioned at the center of a clinical 1.5T and 3.0 T MRI scanners (GE, Signa Excite). Single RF pulses were executed in 1 sec intervals at a constant power output and an RF probe, consisting of two orthogonal single‐loop coils and a shorted coaxial cable (to account for offset voltage), was moved along the z‐axis at 2 cm increments. Induced voltages were measured using an oscilloscope. Measurements were repeated without the RF shield for comparison. RF shield effects on RF‐induced heating was evaluated using fluoroptic thermometers attached to ends of three parallel 40 cm straight copper wires submerged 20 cm apart inside the ASTM head‐and‐torso phantom (Fig. 1(b)). Temperature history during execution of high SAR RF pulse sequence was recorded with and without RF shield around the phantom. The heating of the RF shield itself was measured by 5 fluoroptic thermometers placed at various positions on the surface of the shield.

### Results

At 1.5T, the RF attenuation inside the RF shield increased from 5 dB near the edges to more than 10 dB at 9 cm from the edges, exceeding 30 dB at the center of the shield. At 3.0T, the RF attenuation reached 30 dB at the center of the shield. This attenuation was sufficient to reduce wire heating below levels detectable by fluoroptic thermometry. A measurable amount of heating of the shield was observed, which varied depending on position, but was less than 1.3°C.

### Conclusion

The RF attenuation from the shield and the resulting reduced RF heating of metallic wires are significant and merit further evaluation.

**PO‐BPC‐Exhibit Hall‐08**

## Investigation of a Technique for eliminating of Chemical Shift Artifacts with T1‐Weighted FlAiR images at 3.0T in Thoracic Spine imaging examination

E Lavdas^1^ P Mavroidis^2*^ K Vassiou^3^ V Roka^4^ I Fezoulidis^5^ M Vlychou^5^

*Techological Education Institute of Athens,^1^ Athens, Greece, University of Texas Health Science Center (UTHSCSA),^2^ San Antonio, TX, USA and Karolinska Institutet and Stockholm University, Sweden, University of Thessaly,^3^ Larissa, Greece, Health Center of Farkadona,^4^ Trikala, Greece, University Hospital of Larissa,^5^ Larissa, Greece*

### Purpose

The goal of this study is to assess the effect of chemical shift artifacts and fat suppression between contrast‐enhanced T1‐weighted fast spin‐echo (FSE) sequence with fat suppression and contrast‐enhanced T1‐weighted fluid attenuated inversion recovery (FLAIR) sequence with fat suppression in magnetic resonance imaging (MRI) of the thoracic spine at 3.0T.

### Methods

40 patients underwent thoracic spine imaging with clinical indication of suspected intraspinal metastatic disease. T1‐weighted FSE (TR/TE/ETL/NSA:680/17 ms/2/2,matrix size=384×224,scan time=2.36 min) and fast T1‐weighted FLAIR (TR/TI/TE/ETL/NSA:3200/1150/29 ms/7/2,matrix size=448×224,scan time=3.13 min) sequences were compared in the sagittal plane, during the same imaging session on 3.0T MR system using by synergy body phase‐array surface coil. Qualitative analysis between T1‐weighted FLAIR sequence and T1‐weighted FSE for the extent of chemical shit artifact was performed using a scale of 1–5 (1=no artifacts; 2=minimal artifacts; 3=minor artifacts; 4=major artifacts; 5=image not diagnostic due to artifacts).

### Results

With respect to the results of the qualitative analysis, T1‐weighted FSE sequence showed image quality deterioration of 3.0–3.2 (SD:1.7) averaged over the total patient population, while in T1‐weighted FLAIR sequence, image quality deterioration was 1.7 (SD:1.2). More specifically, T1‐weighted FLAIR sequence is scored to be significantly better than T1‐weighted FSE sequence. Due to chemical shift artifacts in the T1‐weighted FSE, 14 of the patients were found to be of nondiagnostic value. On the contrary, in 11 of those 14 patients, no chemical shift artifacts were observed in the T1‐weighted FLAIR sequence. Regarding the efficiency of fat suppression, both sequences achieved successful fat suppression.

### Conclusions

The present study provides evidence that postcontrast T1‐weighted FLAIR sequence with fat suppression may be considered superior to postcontrast T1‐weighted FSE sequence with fat suppression in thoracic spine at 3.0T because it can resolve the problem of chemical shift artifacts that are likely to appear.

**PO‐BPC‐Exhibit Hall‐09**

## Accuracy evaluation for Six‐dimensional Patient Position Detection Using an orthogonal oBi KV image Pair of a Truebeam linear Accelerator

Michael LM Cheung^1*^ KY Lee^1^ YK Ngar^1^ KH Yu^1^ Anthony TC Chan^1,2^

*Department of Clinical Oncology,^1^ Prince of Wales Hospital, Hong Kong SAR, China, State Key Laboratory in Onology in South China,^2^ Chinese University of Hong Kong, Hong Kong SAR, China*

### Purpose

To evaluate the accuracy of six‐dimensional (6D) patient position detection using an in‐house developed software based on a pair of orthogonal kV images taken using the on‐board‐imager (OBI) of a Varian Truebeam linear accelerator.

### Methods

An in‐house software was developed to detect the 6D position (lateral, longitudinal, vertical, pitch, row, yaw) of a patient on the treatment couch by performing 2D–3D image registration between a pair of orthogonal OBI kV images and the planning CT of the patient. A 6D simulation phantom designed by Ngar et.al. (2012) was used for the evaluation of the accuracy of 6D position detection of the software developed. The phantom was scanned using a CT simulator with its 6D shift set to zero. The phantom was then placed on the couch of a Truebeam linac. 20 sets of randomly generated known nonzero 6D shift with magnitudes within 20 mm and 4° were applied to the phantom. A pair of orthogonal OBI kV images was taken at OBI angles 45° and 315°, respectively, for each set of 6D shifts. The CT and the OBI images were imported into the in‐house developed software for 6D position detection. The detection error was obtained by subtracting the actual shift from the software detected shift.

### Results

The detection error of the 6D positions of the 20 sets of nonzero 6D shifts of the phantom detected were found to be all within 1 mm and 1°. The mean translation detection error and mean rotation detection error were 0.68 mm and 0.56°, respectively.

### Conclusion

With the help of a proper image registration software, a Truebeam linear accelerator was able to perform 6D patient position detection with an accuracy of <1 mm and <1° using an orthogonal OBI kV image pair.

**PO‐BPC‐Exhibit Hall‐10**

## Impact of Protocol Change in the Cardiac Cath lab

L Page^*^ G Tomakin, A Jones

*MD Anderson Cancer Center, Houston, TX*

### Purpose

To quantify the impact on dose to patients and personnel in the cardiac cath lab as a result of increasing the dose per frame for cine acquisition from 0.17 to 0.24 microGy/frame at the request of a physician.

### Methods

Reports containing the dose area product (DAP) and reference air kerma (Ka,r) for patients who underwent left heart catheterization (LHC, n=184) and right and left heart catheterization (RLHC, n=100) procedures on a biplane Siemens Artis Zee were collected. Dose metrics from procedures performed before and after the protocol change were compared. Occupational dose reports were also analyzed.

### Results

After the protocol change, the DAP and Ka,r increased by 3% and 4%, respectively, for LHC. These changes were not significant (p=0.881,0.851). The DAP and Ka,r for RLHC increased significantly by 59% and 64% (p<0.001 for both). These increases were greater than expected based on the change made to the protocol. A review of fluoroscopy time (FT) data revealed that FT for LHC was the same before and after the change, while FT for RLHC increased significantly (35%, p=0.008). Occupational dose data were of limited utility because of inconsistent use of radiation badges.

### Conclusions

Patient dose metrics increased for RLHC procedures but did not increase for LHC procedures after increasing the dose per frame for cine. The constancy of LHC dose metrics may have resulted from a reduction in the number of cine runs acquired owing to improved image quality after the change. The increase in FT for RLHC indicated that another factor was affecting dose metrics, perhaps related to the skill of physicians or the complexity of cases; this is under investigation. It was not possible to assess the impact on occupational dose owing to inconsistent use of radiation badges.

**PO‐BPC‐Exhibit Hall‐12**

## A Simple Commissioning Procedure for a HDR Brachytherapy intracavitary Applicator

A Corrao^1*^ I Mihaylov^2^

*21st Century Oncology,^1^ Providence, RI, Rhode Island Hospital / Warren Alpert Medical,^2^ Providence, RI*

### Purpose

To outline a commissioning procedure for a HDR brachytherapy intracavitary applicator.

### Method and Materials

The described procedure is applied to Nucletrons multicatheter cylinder set with an intravaginal tube and three different intrauterine tandems. The applicators were taken apart and their dimensions were compared to the manufacturer specifications. The index length was measured for all the catheters. All applicators where CT scanned with IU3 markers placed in the central and two outer catheters on opposite sides of the applicator. Applicator sizes on the CT scans were verified. Measurements between the first dummy source and the outer edge of the applicator were performed. Treatment plans for all cylinder and tube/tandem combinations were performed. The plans consisted of a single (first) dwell position in one peripheral catheter and in the tube/tandem. The plans were delivered and autoradiographs taken with a XV film. Prior to delivery, films were marked such that the cylinder and tube/tandem positions and dimensions can be visualized after film processing.

### Results

The index length was measured for all the catheters ‐ the central catheter measured 150 cm, while the outer catheters measured 129.3 cm (per vendor specifications). Cylinder integrity was visually verified in the TPS. The measured autoradiograph distances between the source positions and the cylinder/tube/tandem walls matched the measured distances between the dummy sources and the cylinder/tube/tandem walls in the TPS.

### Conclusion

A simple procedure for HDR brachytherapy applicator commissioning was presented. Verification of the applicators integrity was verified. Coincidence between geometric and dosimetric properties was validated.

**PO‐BPC‐Exhibit Hall‐13**

## CT environmental Surveys: Practical Considerations

G Barnes, R Al‐Senan

*Department of Radiology, University Alabama Birmingham, Birmingham, AL*

### Purpose:

In many states, environmental surveys of new X‐ray installations are required. The most critical in terms of radiation levels to occupationally exposed personnel and the general public are CT installations. Presented are a methodology for doing a thorough and time efficient CT environmental survey, and the results of a survey.

### Methods

The CTDI body phantom is suspended on the CT gantry shroud with its bottom slightly above the table so that the phantom remains stationary as the table moves during an image acquisition. The phantom is suspended by two Lucite bars with adjustable brackets attached to the bottom of the phantom. On a given wall or barrier a fast response hand held scintillation meter is utilized to scan the barrier and determine the area where the highest radiation level occurs. The location is marked with tape. The long CT scan time permitted by suspending the phantom allows one to survey a large barrier area during one scan. Subsequently an exposure rate reading is made at the location with a sensitive ionization survey meter. The readings are normalized to estimate weekly radiation levels. Shielding design Workload was 6,400 mA‐min/wk at 140 kVp. Survey technique was 140 kVp, 100 mA, and 20 sec.

### Results

The readings one foot from the walls, doors, and control window ranged from 0.5 to 5.5 mR/ hr. The associated normalized readings for uncontrolled areas ranged from 0.07 to 1.2 mR/wk. The controlled area normalized readings ranged from 1.5 to 4.5 mR/wk.

### Conclusions

The CT environmental radiation survey was done in a relatively short time (less than two hrs) and was done for a worst case scenario (i.e., body CTDI phantom, and a busy workload at 140 kVp); and for this worst case scenario, the normalized readings are well below regulatory limits for both controlled and uncontrolled areas.

Equipment discussed in this presentation is commercially available from CIRS or at one time was available from Victoreen.

**PO‐BPC‐Exhibit Hall‐14**

## A Comparison of Three Quality Assurance Tools Used for iMRT QA

R Hoffman^*^ T Ivanova, G Cardarelli, K Norton, T Steger, K Dwyer, M Rossi, G Qian, A Jones

*Hartford Hospital, Hartford, CT*

### Purpose

To evaluate IMRT QA plans using three different measurement methods.

### Methods

Three IMRT QA techniques were compared: 1) 2D dose distributions acquired by 2D diode array (MapCHECK2, Sun Nuclear) processed with MapCHECK software; 2) EPID acquired fluences processed in Dosimetry Check (Standard Imaging); and 3) film dose measurements processed by RIT (Radiological Imaging Technology). The IMRT/VMAT plans used in this study included head and neck, prostate, chest, pelvis. The acceptance criteria for the gamma analysis used to evaluate the QA results are 3% dose difference and 3 mm distance to agreement for 90% for all points in the plane or volume being compared. The cases that were chosen for the analysis initially provided less than the desired gamma analysis results using Dosimetry Check (DC). Additionally, each software analysis tool was used to evaluate the three different detectors, 2D diode array, EPID, and film for the same plan.

### Results

For the cases studied, MapCHECK QA analyses passed with at least 95% of points analyzed having gamma less than 1. In all but one case, RIT QA analyses passed with 90% of points analyzed having gamma less than 1.

### Conclusion

IMRT QA analysis based on the planar dose comparison (i.e., MAPCHECK and RIT) provide higher passing rates than DC, which uses volumetric dose comparison (e.g., in the PTV) computed from fluence reconstructed from an EPID projected onto the patient 3D dataset. Results to date demonstrate the following: 1. Of the 3 detector types there was a higher passing rate for film for small field plans. For lower gradient plans, EPID ranked second and 2D Diode array ranked last. 2. For routine plans, MapCHECK is the tool of choice. 3. For complex plans, Dosimetry Check with EPID or film is the desired IMRT QA tool.

**PO‐BPC‐Exhibit Hall‐15**

## Unusual Characteristics of the HD MlC on a TrueBeam STx

A Hsu^*^ E Mok

*Stanford Cancer Center, Stanford, CA*

### Purpose

During acceptance testing of the high definition MLC (HDMLC) on a Varian TrueBeam STx machine, unusual characteristics were noted in its mechanical capability. We present some of those findings and discuss their clinical impact.

### Methods

Picket Fence tests were run on both a Varian TrueBeam STx linac with an HD MLC and a Varian Clinac 21EX with a Millennium 120 MLC (Varian Medical Systems, Palo Alto, CA). Images were acquired with both EDR2 film and portal imaging (EPID).

### Results

The Picket Fence result on a Millennium MLC shows the typical repeating pattern of gaps (dark due to exposure) and pickets (lighter due to the MLC blocking). For the HDMLC, the some of the gaps are much lighter than the pickets. This was observed in both EDR2 film and EPID imaging. There also appears to be nonuniformity in the gaps over the width of the carriage and depending on which MLCs, the small 0.25 cm leaves versus the larger 0.5 cm leaves were being moved. The vendor was contacted and additional testing was done. One hypothesis is that this behavior may be due to the intrinsic backlash of the MLC and is magnified by leaf curvature and the fact that the leaf gap for the TrueBeam is smaller than that for the Millennium MLC. It is also thought that leaf over travel plays an effect. Discussions with the vendor yielded the additional information regarding software controls of the offset of the HDMLC.

### Conclusion

The HDMLC on a TrueBeam is characteristically different than a Millennium MLC on a Clinac 21Ex. Reasons for this include hardware differences of the MLCs and software corrections that are handled by the TrueBeam. However, of primary clinical interest will be how these differences across the field profiles affect dosimetry and the modeling of the leaf gap in the planning system.

**PO‐BPC‐Exhibit Hall‐16**

## Optimization of Dose Distribution for Different Vaginal Cylinders of HDR Brachytherapy

S kumar^*^ A Silpa, C Suja, B Resmi

*Malabar Cancer Centre, Kannur, Kerala*

### Purpose

Optimization of dose distribution for different vaginal cylinders of HDR brachytherapy using different optimization methods, namely iterative, geometric and equal time.

### Methods

Four different vaginal cylinders with diameters ranging from 2.6 to 4 cm is selected and dose reference points on the surface of the cylinders have been defined to optimize a dose of 6 Gy for a treatment length of 6 cm. Three optimization methods available in the TPS have been used to calculate the dose distribution. Apex points at the tip of the dome of the applicator and along the curvature of the dome was defined as dose reference points (DRP), and optimization was repeated with these points included. Comparison has been made between the dose distribution obtained with and without these apex points.

### Results

Whenever we specify apex points as dose reference points, the minimum and maximum variation of dose with respect to the mean dose is higher than the variations compared to calculations made when apex points are not specified. When apex points are defined, the treatment volume increased by 8% and area only by 2% in case of 3 cm diameter cylinder. Treatment volume increases by 14% and area increases by 2% as observed in case of 3.5 cm diameter cylinder, when apex points are included in optimization. But with 4 cm diameter cylinder both the treatment volume and area increases by 9%.

### Conclusion

The dose reference points to be defined at the apex of the cylinder to deliver adequate dose at the dome of the cylinder. Even through the variation in doses between apex points and points along the cylinder is maximum, adequate dose is delivered at the vault thereby reducing the frequency of recurrences in these types of treatments.

**PROFESSIONAL SYMPOSIUM SALON EF**

***REGULATORY AND LEGISLATIVE***

**SA‐A‐Salon EF‐01**

## Are There Roadblocks in the Way to Regulatory or legislative Progress?

L Fairobent

*AAPM, College Park, MD*

AAPM is actively involved in reviewing, monitoring, and commenting on proposed regulations and legislation.

This talk will discuss:

• Medical Physics Licensure
• NRC's proposed revision to 10 CFR Part 35, “Medical Use of Byproduct Material
• The American Medical Isotope Production Act

**SA‐A‐Salon EF‐02**

## Linear Accelerator incident investigation

A Rogers^*^ L Bruedigan

H Watkins

*Texas Department of State Health Services, Austin, TX*

This presentation will describe the State of Texas’ Radiation Control Program's response to a medical misadministration involving the omission of a required conical collimator during a stereotactic radiosurgery treatment.

**THERAPY SYMPOSIUM – SAM SALON EF**

***SMALL FIELD DOSIMETRY FOR SRS, SBRT, IMRT, AND VMAT***

**SA‐B‐Salon EF‐01**

## Small Field Dosimetry for SRS, SBRT, iMRT, and VMAT

I Das,^1*^ D Low^2*^

*Indiana University School of Medicine,^1^ Indianapolis, IN, UCLA,^2^ Los Angeles, CA*

Small fields on the order of few millimeters are now being used in specialized radiation treatments such as intensity‐modulated radiation therapy (IMRT), VAT, stereotactic radiosurgery (SRS), CyberKnife, and Gamma Knife. Small‐field dosimetry is challenging due to finite source size, lack of electronic equilibrium, size of detectors, changes in energy spectrum with associated dosimetric parameters, and stopping power ratio. Poor understanding of dosimetry has lead to many errors and wrong patient treatments. Source size is dependent on the design of the accelerator and could be obstructed by the collimating system with decreasing field sizes. Electronic equilibrium is a phenomenon associated with the range of secondary particles and hence dependent on the beam energy, spectrum, and the composition of the medium (homogeneous vs. inhomogeneous). This talk will elaborate on safety concerns in advanced treatment techniques. It will also provide current understanding of the small‐field dosimetry and give insight of the new IAEA/AAPM noncomplainant dosimetry^(1)^ IPEM Report 103^(2)^ and AAPM Task Group Report 155.^(3)^ Dosimetric criteria as stated in TG‐120^(4)^ will be explored. Explanation of the difficulties and possible solutions to accurately measure dose in small fields with various techniques (ion chamber, diode, diamond, TLD, films (radiographic and GAFCHROMIC), MOSFET, bang gel, and Monte Carlo) will be elaborated. Correction factors that have been published will be presented and the consequences in accuracy for patient care will be discussed.

### Learning objectives

1. Devices that deals with small fields.
2. Definition of small fields.
3. Description of absolute and relative dosimetry.
4. Choice of detector.
5. Correction factor.
6. Understanding the implication of small fields in clinical practice.

**DIAGNOSTIC SYMPOSIUM – SAM**

**SALON BCD**

***CT: SSDE / NEW ACR QC MANUAL***

**SA‐B‐Salon BCD‐01**

## CT

D Pfeiffer

*Boulder Community Foothills Hospital, Boulder, CO*

The American College of Radiology Computed Tomography Quality Control Manual was published on 12/1/2012. This manual, which must be followed by facilities accredited by the ACR, describes the roles of radiologists, technologists, and medical physicists. It specifies quality control tests to be performed by technologists and medical physicists. This presentation will describe the QC manual, highlighting responsibilities of the team members. The required technologist and medical physicist tests will be described. Further developments around the QC Manual and the CT Accreditation Program will also be discussed.

### Learning Objectives

1. Understand the responsibilities of the various team members as described in the manual.
2. Learn the technologist quality control tests.
3. Learn the medical physicist quality control tests.
4. Be aware of errata published since the original publication of the manual.

**SA‐B‐Salon BCD‐02**

## CT: Size‐Specific Dose Estimate (SSDE): Why We Need Another CT Dose index

K Strauss

*Cincinnati Children's Hospital Medical Center, Cincinnati, OH*

Historically, two CT dose indexes, CTDIvol and Dose Length Product (DLP), have been used to quantify the delivery of radiation dose to standardized phantoms by the CT scanner during a clinical examination. Too many radiologists, technologists, and even some medical physicists do not realize that neither dose index is a reasonable estimation of the patient's radiation dose during the examination. The Size‐Specific Dose Index (SSDE) was recently developed by AAPM Task Group 204 to address this need for all size patients, from the smallest child to the largest adult. This lecture will begin by defining CTDIvol, DLP, and Effective Dose (E Dose), and then explore why none of these dose estimates are reasonable estimates of patient dose.

SSDE will be defined and explained based on the task group's published report. Sample calculations of estimated patient doses based on SSDE will be provided. Useful clinical applications of SSDE will be explored, such as dose reporting in the patient's medical record, eliminating confusion about CT doses from dissimilar CT scanners, and first estimates of organ doses from SSDE calculations. A method using SSDE will be explained that allows the consulting medical physicist to develop appropriate CT radiographic techniques for any CT scanner in any department, from the smallest pediatric patients to the largest bariatric adults. The presentation will conclude with a description of challenges and solutions associated with effectively using the scanner's Automatic Exposure Control feature during pediatric imaging.

### Learning Objectives

1. Understand the inappropriateness of using CTDIvol, DLP, or E Dose to estimate patient radiation dose during CT scanning.
2. Understand the strengths and weaknesses of SSDE as an estimate of patient radiation dose during CT scanning.
3. Understand how SSDE can be used in the clinic to develop appropriate CT radiographic techniques for any CT scanner in any clinical department.
4. Understand the basic problems and potential solutions to proper utilization of the Automatic Exposure Control features of a CT scanner for pediatric imaging.

**SUNDAY, MARCH 17**

**THERAPY SYMPOSIUM – SAM**

**SALON EF**

***FOUNDATIONS OF RADIATION PHYSICS: MONITOR UNIT CALCULATIONS***

**SU‐A‐Salon EF‐01**

## Foundations of Radiation Physics: Monitor Unit Calculation and Verification

J Gibbons^1*^ R Stern^2*^

*Mary Bird Perkins Cancer Center,^1^ Baton Rouge, LA, UC Davis Cancer Center,^2^ Sacramento, CA*

AAPM Task Group 71 has defined the nomenclature and methodology for performing monitor unit (MU) calculations for photon and electron beams. Calculations within this protocol are made using the dose per MU under normalization conditions that is determined for each user's beams. For both photon and electron beams, this normalized dose per MU and associated dosimetric functions are determined using flat, water phantom data. The requirement of an independent verification of the monitor units calculated to deliver the prescribed dose to a patient has been a mainstay of radiation oncology quality assurance. However, in a modern clinic using CT/MR/PET simulation, computerized 3D treatment planning, heterogeneity corrections, and complex calculation algorithms such as convolution/superposition and Monte Carlo, the purpose of and methodology for the MU verification have come into question. In addition, since the verification is often performed using a simpler geometrical model and calculation algorithm than the primary calculation, guidelines are needed to help the physicist set clinically reasonable action levels for agreement. The AAPM Task Group 114 report reevaluates the purpose and methods of the “independent second check” for monitor unit calculations for non‐IMRT radiation treatment in light of the complexities of modern day treatment planning.

The report provides recommendations on how to perform verification of MU calculations in a modern clinic, and on the establishment and implementation of action levels for agreement between primary calculations and verification.

### Learning Objectives

1. Understand the current‐recommended nomenclature, methodology, and measurements required to perform MU calculations within the TG71 protocol.
2. Know the components of a good monitor unit verification program.
3. Be able to apply the action level guidelines for agreement between primary and verification monitor unit calculations presented in the TG114 report.

**MAMMOGRAPHY SYMPOSIUM**

**SALON BCD**

***ADVANCED MODALITIES IN BREAST IMAGING ‐ MOLECULAR BREAST IMAGING AND STEREOTACTIC BREAST BIOPSY***

**SU‐A‐Salon BCD‐01**

## Advanced Modalities in Breast imaging / Molecular Breast imaging and Stereotactic Breast Biopsy

M Martin

*Therapy Physics, Inc., Gardena, CA*

The performance of the 11 required physics tests on a Stereotactic Breast Biopsy (SBB) System as required for accreditation by the American College of Radiology (ACR) will be covered in this presentation covering systems both with and without Automatic Exposure Control Systems for both prone biopsy tables and add‐on SBB units for digital FFDM units. Image quality, expected values for half‐value‐layer (HVL), and patient doses for each matrix size available on SBB systems will be discussed. Clinical advantages/disadvantages of both types of systems will be covered relative to approach methods and areas available for sampling. The importance of testing the location of the biopsy needle or tissue sampling device for reproducibility and accuracy will be discussed relative to various phantoms that may be used to verify this location accuracy. Quality control (QC) tests to be performed by the mammography technologist will be covered in addition to the physics required tests. Requirements for training and education of the staff (physician, physicist, and technologist) so as to achieve accreditation by the ACR will be reviewed now that there is a requirement for SBB units to be accredited for breast imaging centers in order to obtain the ACR designation of a Breast Imaging Center of Excellence.

### Learning Objectives

1. Attendees will be aware of the required physics tests to be performed annually and for acceptance of Stereotactic Breast Biopsy (SBB) System.
2. Attendees will be aware of the expected values of half‐value layer and patient doses for SBB systems for common matrix sizes available on these units.
3. Attendees will be aware of the requirements for personnel performing SBB exams or physics evaluations in both Continuing Education and Continuing Experience for accreditation by the American College of Radiology.
4. Attendees will be aware of the required quality control tests to be performed by both the technologist and physicist on SBB units.

**SU‐A‐Salon BCD‐02**

## Establishing a New Breast‐Specific Gamma Imaging (BSGi) Program: Roles of the Medical Physicist

J Clements

*Texas Health Presbyterian Hospital Dallas, Dallas, TX*

Breast‐Specific Gamma Imaging (BSGI) is a molecular breast imaging (MBI) procedure using a high‐resolution and small field‐of‐view gamma camera. In addition to breast‐specific imaging, the camera may be used for general nuclear medicine procedures. This presentation will update medical physicists of their responsibilities for supporting this system, including a review of the technology and clinical advantages, regulatory aspects for radioactive materials use, acceptance and quality control testing procedures, and how to obtain accreditation.

### Learning objectives

1. Review Breast‐Specific Gamma Imaging (BSGI) technology and clinical advantages.
2. Understand radioactive materials licensing issues and training for staff and physicians.
3. Learn about medical physicist acceptance testing and quality control procedures.
4. Understand the process to receive accreditation.

**THERAPY SYMPOSIUM ‐ SAM**

**SALON EF**

***FOUNDATIONS OF RADIATION PHYSICS: EBRT QA***

**SU‐B‐Salon EF‐01**

## Foundations of Radiation Physics: external Beam Radiation Therapy Quality Assurance

S Kry^1*^ F Yin^2*^ Y Xiao^3*^

*MD Anderson Cancer Ctr.,^1^ Houston, TX, Duke University Medical Center,^2^ Durham, NC, Thomas Jefferson University Hospital,^3^ Philadelphia, PA*

This session will cover three aspects of external beam therapy, focusing on quality assurance.

1. TG‐142: Quality assurance of medical accelerators.
2. Flattening filter‐free (FFF) C‐arm linear accelerators.
3. Volumetric‐modulated arc therapy.

The first section will review the principles of conducting QA for linear accelerators and discuss topics related to the execution of QA tasks, such as measurement parameters, measurement method, frequencies, tolerances, actions, documentation, measurement tools, staff, and effort. Examples of TG‐142 QA implementation in a specific institution will be illustrated. Limitations of TG‐142 and challenges will be discussed, as well. The second section will provide a technological review of flattening filter‐free beams. It will also cover acceptance testing, commissioning, and periodic quality assurance for these beams. Finally, clinical applications and limitations of flattening filter‐free beams will be highlighted. The third section will discuss acceptance testing and commissioning for VMAT. It will also address routine quality assurance procedures for VMAT, including machine‐specific and patient‐specific QA. Finally, it will discuss issues relating to VMAT implementation in a clinical environment, including identifying when VMAT may improve efficiency, as well as limitations and cautions in treatment planning and the delivery process when VMAT technology is utilized.

### Learning objectives

1. Understand the principle and content of TG‐142, as well as a strategy and methodology of implementing the recommendations in this report.
2. Understand the limitations of TG 142.
3. Understand the techniques and challenges of commissioning and maintaining flattening filter‐free beams.
4. Understand the process of implementing a VMAT program.
5. Understand the clinical strengths and weaknesses of FFF and VMAT techniques.

**MAMMOGRAPHY SYMPOSIUM ‐ SAM**

**SALON BCD**

***DIGITAL BREAST TOMOSYNTHESIS: BASIC PHYSICS AND OPERATION***

**SU‐B‐Salon BCD‐01**

## Digital Breast Tomosynthesis: Basic Physics and operation

L Greer

*Phoenix, AZ*

This lecture is for the purpose of introducing the audience to digital breast tomosynthesis: the advantages of this technology, how images are obtained and reconstructed, how it has changed daily mammography practice, and radiation dose issues. We will also be introducing C View, which is currently under FDA review. Examples will be shown of 2D vs. 3D mammograms, demonstrating marked improvement in cancer detection. I do lecture on behalf of Hologic, but stipends for these lectures are paid to JC Lincoln Health Foundation, and I personally receive no money for these services. There are no known conflicts of interest.

**SU‐B‐Salon BCD‐03**

## History, Theory and operation of Digital Breast Tomosynthesis Systems

M Goodsitt,^*^

H Chan

*University Michigan, Ann Arbor, MI*

Tomosynthesis is a quasi 3D tomographic X‐ray imaging method whereby a set of slices are reconstructed from a series of projection images acquired over a limited angle. Techniques using film as the detector were invented in the 1930s and application to the breast using digital detectors (digital breast tomosynthesis (DBT)) began in the 1990s. A wide variety of DBT systems presently exist, but only one system has been approved by the FDA for clinical use, to date. The current DBT systems will be reviewed, emphasizing the major design and operational differences. Image quality in DBT depends upon many factors including the acquisition geometry (total tomographic angle and angle increments), the accuracy of the geometric parameters used in reconstruction, X‐ray tube motion during the exposures, and the reconstruction method. These and other factors will be discussed. Several types of artifacts in reconstructed DBT images will also be described. Finally, new applications such as contrast‐enhanced DBT and dual‐modality DBT will be examined.

### Learning objectives

1. Understand the basic theory of tomosynthesis image generation (the shift and add principle).
2. Understand the effects of different imaging geometries (e.g., narrow tomographic angle vs. wide tomographic angle) on the perception of masses, microcalcifications, and contrast‐detail test objects in DBT images.
3. Understand the origins of common tomosynthesis artifacts.

NIH R01 (PI: Carson, Paul) in collaboration with GE Global Research, Title: Combined Digital X‐Ray Optical and Ultrasound Breast Imaging; NIH R01 (PI: Chan, Heang‐Ping) in collaboration with GE Global Research, Title: Improvement of Microcalcification Detection in Digital Breast Tomosynthesis.

**PROFESSIONAL SYMPOSIUM**

**SALON EF**

***ABR TRUSTEES AND CONTINUOUS CERTIFICATION & PQI***

**SU‐C‐Salon EF‐01**

## ABR Trustees and Continuous Certification & PQI

R Morin

*Mayo Clinic, Jacksonville, FL*

This presentation will discuss the aspects of the American Board of Radiology, Maintenance of Certification (MOC) process. In addition, the new process of continuous certification will be explained and clarified, including the ramifications of proper and timely completion of each part of MOC.

### Learning objectives

1. Review the American Board of Medical Specialties MOC process.
2. Review the ABR MOC process.
3. Review the specifics for Medical Physics MOC.
4. Explain the continuous certification process.

**SU‐C‐Salon EF‐02**

## Medical Physics Practice Quality improvement Guidelines

M Yester

*UAB Medical Center, Birmingham, AL*

Currently, a common initiative in many fields is quality improvement. This endeavor is especially prominent in the medical community with concerns of patient safety and reduction of medical errors. As part of its certification oversight, the American Board of Medical Specialties (ABMS) has made QI one of the four sections of its Maintenance of Certification (MOC) process. This is particularly relevant to those medical physicists with time limited ABR certificates or for other ABR Diplomates voluntarily enrolled in the MOC program. One of the key aspects of The ABR expectation for Practice Quality Improvement (PQI), Part IV of the ABR MOC program, is that Diplomates provide evidence of an ongoing program of improvement of practice, either as an individual or within the system the individual is employed. For physicists, this may seem somewhat nebulous due to the many duties and responsibilities for quality in the clinical realm. For diagnostic physicists, this may seem even more undefined, especially for consultants. As a beginning dialogue related to PQI, suggestions for projects appropriate for medical physicists are presented. Another method for fulfillment of the PQI section is participation in society based PQI programs. Such programs are under development within AAPM and formulations of a program for physicists is presented.

### Learning objectives

At the conclusion of the presentation, an individual will:

1. Gain knowledge of the basic aspects of PQI as regards to project types, basic ingredients of projects.
2. Learn about examples of projects for demonstration of PQI for medical physicists.
3. Learn about the developments of society‐based program for PQI for Physicists within the AAPM.

**PROFESSIONAL SYMPOSIUM ‐ SAM**

**SALON BCD**

***PROFESSIONAL LIABILITY AND RISK MANAGEMENT IN MEDICAL PHYSICS PRACTICE***

**SU‐C‐Salon BCD‐01**

## Anatomy of a Professional liability Case

J Masten

*Aspirus Regional Cancer Center, Wausau, WI*

### Purpose

To introduce the meeting participants to the basic terminology and structure of medical negligence litigation. This talk will deal in depth with the tools which lawyers use to shape the case, the stage at which a particular tool is available, and the reasons behind a particular approach.

### Methods

Actual case law will be used to illustrate the various legal concepts. Secondary legal sources will be introduced, as well as an extensive handout to point the way for further study.

### Conclusions

Session participants should come away with not only an understanding of the language of litigation, but also a basic appreciation of the strategy in civil litigation procedure and its significance to the outcome of a trial.

**SU‐C‐Salon BCD‐02**

## Liability Risk Management for Practicing Medical Physicists

D Jordan

*University Hospitals Case Medical Center, Cleveland, OH*

The risks of financial harm to medical physicists due to professional liability are complex and often difficult to determine clearly. This presentation introduces the terminology and concepts of professional liability as it relates to the practice of clinical medical physics. Physicists will learn about situations that would lead to claims for financial damages under professional liability. The terminology of professional liability insurance will be introduced, enabling physicists to understand how insurance works and to determine the adequacy of a particular product for their individual needs. An approach to determining the need for coverage, as well as the amount of coverage, will be discussed.

### Learning objectives

1. Identify risks and liabilities arising from the practice of medical physics.
2. Explain the role of professional liability insurance in a medical physicist's risk management.
3. Understand terms and conditions typically encountered in liability insurance policies.
4. Determine the amount and type of coverage needed to protect personal assets from uncovered or inadequately covered risks.

**THERAPY SYMPOSIUM ‐ SAM**

**SALON EF**

***QA OF TREATMENT GUIDANCE SYSTEMS***

**SU‐D‐Salon EF‐01**

## QA of Treatment Guidance Systems

T Willoughby,^1*^ B Salter,^2*^ J Bissonnette^3*^

*M.D. Anderson Cancer Ctr Orlando,^1^ Orlando, FL, USA, University Utah,^2^ Salt Lake City, UT, USA, Princess Margaret Hospital,^3^ Toronto, ON, Canada*

Image‐guidance radiation therapy (IGRT) systems are now widely available and implemented for routine clinical use in many clinics, in large part due to their tight integration with medical linear accelerators and treatment planning systems (TPS). Increased geometric precision and accuracy are now widely achievable in radiotherapy delivery because users can correct patient translations and rotations immediately prior to administration of radiotherapy by registering IGRT ‘images of the day’ to those initially used for treatment planning. Furthermore, comparison of successive daily IGRT images can help to quantify changes of internal anatomy throughout the course of therapy. However, introducing image guidance within busy radiation therapy clinics requires thoughtful commissioning and quality control (QC) protocols, and judicious modification of existing radiation therapy processes and protocols. While the uses of such novel systems will continue to evolve, their performance must be diligently maintained at the highest level, as they will be relied upon to insure accuracy in the treatment delivery process. In fact, such systems may be used to justify PTV margin reduction, thus further emphasizing the need for accuracy and precision through careful QC. There are two key features of IGRT systems that require particular attention: geometric accuracy and image quality. First, as a separate modality, the chosen IGRT system possesses its own reference origin and coordinate system, which in some cases may not be physically coincident with that of the megavoltage treatment beam or the medical linear accelerator isocenter. Therefore, the geometric relationship between the IGRT dataset and the megavoltage treatment beam must be established and monitored to ensure adequate localization, coincidence, orientation, and scaling. Second, image quality ultimately determines the ability of the IGRT system to consistently produce images capable of localizing the structures of interest, through either manual or automatic registration. Careful and continual monitoring of image quality must be performed to ensure consistent and accurate image guidance results. Details of a QC program for various IGRT solutions will be highlighted based on the following reports: TG147: Non‐Radiographic Systems for Localization, TG154: Ultrasound Guidance Systems for External Beam Radiotherapy for Prostate Cancer, TG179: External Beam Image Guidance using CT‐based Technologies.

### Learning objectives

1. To present IGRT technologies covered by the referenced Task Groups and major considerations for their uses.
2. To describe principles for quality control of IGRT systems.
3. To characterize and discuss the impact of IGRT on quality and safety of radiation therapy.

Jean‐Pierre Bissonnette has a commercial interest with Modus Medical.

**MAMMOGRAPHY SYMPOSIUM ‐ SAM**

**SALON BCD**

***DIGITAL BREAST TOMOSYNTHESIS ACR/MQSA REQUIREMENTS AND FACILITY DESIGN***

**SU‐D‐Salon BCD‐01**

## Update on MQSA and ACR Mammography Accreditation Program

P Platt

*American College of Radiology, Reston, VA*

### Learning objectives

The presentation will enable participants the opportunity to acquaint themselves with the rules under the Mammography Quality Standards Act (MQSA) governing mammography facilities and accrediting bodies (AB) as it applies to digital breast tomosynthesis (DBT). Additionally, an update will be provided on the ACR's Breast Imaging Center of Excellence Designation. The participants will be provided an overview of the ACR accreditation / MQSA certification process to:

1. Understand the difference between accreditation and certification under the Mammography Quality Standards Act (MQSA); to include the role of the accrediting body, MQSA, the facility, and the annual MQSA inspection.
2. Understand the accreditation process for a facility to start performing mammography as a new facility, or accredited facility adding a new DBT unit(s).
3. Provide an overview of the MQSA Certificate Extension Program for DBT.
4. Understand the medical physicist role as a member of the mammography team in providing guidance to the facility both during and after the annual survey.
5. Provide guidance on where to go for assistance.
6. Provide an overview of the ACR Breast Imaging Centers of Excellence.

### Outline

1. Accreditation and Certification
  - MQSA
  - Accrediting Bodies
    - ACR
    - Arkansas
    - Iowa
    - Texas
  - MQSA/State Inspectors
2. ACR Accreditation Process for New Units with An Accrediting Body
  - New units
  - New facilities
  - Needed documentation
    - Accreditation application
    - Medical Physicist Mammography Equipment Evaluation
  - Common Application Mistakes
3. Why No Accrediting Body
  - Newly Approved FFDM Systems
    - All ABs must apply to be an accrediting body to the FDA
  - New Modality
    - Need an established standard of care
4. Accreditation Process for New Units without An Accrediting Body – MQSA Certificate Extension Program for DBT
  - Process
  - Needed Documentation
    - Facility information
    - Unit information
    - List of qualified personnel
    - Medical physicist mammography equipment evaluation; including phantom image
  - FDA Approval Letter
5. FDA Annual Inspection
  - AB Accredited Units
  - MQSA Certificate Extension Units
6. Hologic Lorad Selenia Dimensions Information
  - Facility Information
  - Consumer Information
7. Where to go for help
  - Medical Physicist
  - FFDM Unit Manufacturer
  - MQSA Policy Guidance Help System
  - ACR Website
  - Hologic Website
8. ACR Breast Imaging Centers of Excellence
  - Criteria for designation
  - Statistics

### Conclusion

Course participants should demonstrate an understanding of the basic principles of accreditation and certification under MQSA, to include the units that have an accrediting body and those units that fall under the MQSA Certificate Extension Program; to include common pitfalls during the application process and where to go for assistance. Additionally, participants will be able to demonstrate an understanding of the ACR's Breast Imaging Centers of Excellence designation.

**SU‐D‐Salon BCD‐02**

## Tomosynthesis from the Ground Up

W Geiser

*UT MD Anderson Cancer Center, Houston, TX*

Breast tomosynthesis was approved for use in screening for breast cancer in February 2011. Many tomosynthesis ready systems were installed across the country in preparation for this approval and those systems are being upgraded to perform Digital Breast Tomosynthesis (DBT). There is evidence that DBT is helping reduce recall rates while increasing cancer detection rates.^1^, ^2^, ^3^ Due to this evidence, many new systems are also being installed. Facilities that are implementing a breast tomosynthesis program need to be aware that there are many hurdles that need to be overcome before tomosynthesis studies can be performed. The purpose of this lecture is to give the medical physicist the knowledge needed to help those facilities that are going to perform DBT deal with facility design issues, image storage requirements, choice of PACs, and review of the studies. This will include a review of DICOM for DBT, shielding requirements for DBT suites, and choice of review stations to help facilities transition smoothly form reading of 2D studies to reading of combined 2D and 3D studies.

### Learning objectives

1. Understand shielding requirements for a breast tomosynthesis suite.
2. Be able to make recommendations to facility managers on PACs and review station requirements for breast tomosynthesis.
3. Be able to help facility managers with implementation of breast tomosynthesis at their facility.

**MONDAY, MARCH 18**

**THERAPY SYMPOSIUM ‐ SAM**

**SALON EF**

***ADVANCES IN BRACHYTHERAPY DOSE CALCULATIONS***

**MO‐A‐Salon EF‐01**

## Advances in Brachytherapy Dose Calculations

L Beaulieu,^1*^ F Mourtada^2*^

*Centre Hospitalier University de Quebec,^1^ Quebec, QC, Canada Christiana Care Hospital,^2^ Newark, DE, USA*

Recent advances in dose calculation algorithms for brachytherapy will have profound impact on how the end users will be required to integrate the necessary new information into the treatment planning system (TPS), commission a TPS that integrate model‐based dose calculation algorithms (MBDCA) and dose reporting requirements. Until recently, all clinical TPS followed the well‐known TG‐43 dose calculation protocol. It is now possible for medical physics to take into account the complete geometry of a treatment (i.e., finite patient boundary (breast, superficial application)) and the heterogeneous tissue composition and any nonwater applicators, shields or other devices. The recently‐published AAPM TG‐186 report discusses important issues for clinical implementation of these algorithms. We will review the motivation leading to TG‐186 work, and present in a clinically oriented fashion the key point of TG‐186 recommendations. In particular, specific examples on how to perform the commissioning process will be presented. A proposed commissioning flowchart will be discussed, guiding the audience through the clinical process, with pitfalls identified to minimize likelihood for errors.

### Learning objectives

1. Identify key clinical applications needing advanced dose calculation in brachytherapy.
2. Provide an overview of the alternatives to TG43.
3. Explain in practical terms the recommendations of TG186.
4. Identify relevant commissioning processes for safe clinical integration.
5. Provide practical clinical examples using a commercially available system.

Luc Beaulieu holds a research agreement with Elekta/Nucletron regarding advances dose calculation algorithms.

Luc Beaulieu and Firas Mourtada are members of an AAPM sponsor Working Group (WG) on model‐based brachytherapy dose calculation. This WG is working closely with all brachytherapy treatment planning system vendors.

**DIAGNOSTIC SYMPOSIUM ‐ SAM**

**SALON BCD**

***MR: MULTI‐VENDOR TESTING / AUTOMATED PHANTOM QC ANALYSIS***

**MO‐A‐Salon BCD‐01**

## Experiences in ACR MRi Accreditation ‐ Vendor nuances That every Clinical MRi Physicist Should Know

K Huff

*Riverview, FL*

Given the multitude of manufacturers that produce magnetic resonance scanners, it is challenging for the MRI physicist/MRI scientist to maintain a working knowledge of every system and its idiosyncrasies. Each vendor, magnet, and software package has its own limitations. Knowing which is a true limitation of the system and what constitutes a genuine artifact or problem can save time in the troubleshooting process. Additionally, understanding the ACR MRI Accreditation phantom, its tests and failures, can provide insight into the overall performance of an MRI scanner. Data collected on more than one thousand scanners established certain general trends. This presentation is a compilation of the experience gained from the testing and problem‐solving of virtually every make and manufacturer in MRI today. The intent of this lecture is to better equip the MRI Physicist/ MRI Scientist to efficiently troubleshoot issues with each type of MR imaging system.

**MO‐A‐Salon BCD‐02**

## Development of An Automated MR Quality Control Program in Compliance with American College of Radiology (ACR) Accreditation Requirements

K McGee,^*^ S Stiving, Z Bao, D Lanners, T Peterson, R Jonsgaard

*Mayo Clinic, Rochester, MN*

In 2008, the Medicare Improvement for Patients and Providers Act (MIPPA) was signed into US public law. Amongst its many provisions, the new law required that all providers of CT, MRI, breast MRI, nuclear medicine and PET examinations that bill under part B of the Medicare Physician Fee Schedule be accredited by January 1, 2012 with noncompliance resulting in the loss of Medicare reimbursement for these services. Since that time, this requirement has been adopted by at least one major private insurance provider. The insistence of accreditation in return for payment by both government and private payers has resulted in the widespread adoption of accreditation of advanced imaging facilities within the US. By far the largest accreditation program is that run by the American College of Radiology (ACR) and includes two components: 1) an initial application that involves the submission of both clinical diagnostic and phantom image data, and 2) following successful accreditation, the establishment of a routine quality control program involving calculation, on a weekly basis, of a variety of metrics from MR images of an ACR‐designed image quality phantom. While the ACR provides detailed methods for manually calculating these metrics, they are time‐consuming, and prone to human bias and error. In addition these measurements can take tens of minutes. For centers with more than one MR scanner, performing these quality control checks can be a significant time constraint for technologists and other support staff. To address this, we have developed an automated, web‐based quality control program. The program involves daily acquisition of ACR MR phantom data, transfer of the data to a remote workstation, processing of these images to extract ACR recommended quality control metrics, and storage of these data in an institutional relational database. Data are then reviewed remotely through an in‐house quality control web application. The web application indicates whether or not each test is within user‐defined limits, thereby determining the pass/fail status of the test. Manual data entry is allowed in accord with ACR requirements. The system was first introduced into our clinical practice in 2006, starting with eight MR scanners and since that time has been expanded to include 38 MR scanners located at the three campuses of the Mayo Clinic (Rochester, MN, Scottsdale, AZ, and Jacksonville, FL). To date, a total of 59,775 phantom studies at field strengths of 1.5T and 3.0T from two MR scanner manufacturers have been processed by this tool. In addition, the application has been successfully commercialized and is offered on a fee‐for‐service basis by an independent startup company.

### Learning objectives

1. Describe the process of developing this system.
2. Describe the pitfalls and challenges encountered.
3. Identify the value of such an approach given the increasing financial constraints experienced by many radiology departments.
4. Provide examples of system detected failures and their root cause.
5. Describe the commercialization process.

Recommendations will be provided for those wishing to develop similar systems for compliance with ACR accreditation recommendations.

**YOUNG INVESTIGATOR SYMPOSIUM SALON EF**

**MO‐B‐Salon EF‐01**

## A Rapid 1D *in vivo* Dose Measurement Technique for VMAT Treatments Using Single integrated Portal images

J Roberts,^1*^ W Ansbacher^2^

*University of Victoria,^1^ Victoria, BC, BC Cancer Agency ‐ Vancouver Island Centre,^2^ Victoria, BC, Canada*

### Purpose

To develop a rapid, accurate *in Vivo* dose measurement technique for use in volumetric‐modulated arc therapy (VMAT) treatments using single arc‐integrated electronic portal images.

### Methods

The dose measured along axis of gantry rotation can be directly related to the signal along the y‐axis of EPIs in integrated mode. A simple back‐projection algorithm was developed using a composite transmission factor averaged over the gantry angles subtended by the treatment arc. Corrections were applied in order to account for beam hardening, scattered radiation within the EPID, phantom‐to‐EPID scatter, and phantom scatter. Four clinical prostate VMAT treatment plans were exported for delivery to an anthropomorphic phantom, and integrated EPIs were recorded.

### Results

Portal image reconstructed doses at isocenter agreed with treatment planning system calculations (mean difference of 4.4% and standard deviation 2.7%). 1D dose profiles along the axis of gantry rotation showed good agreement with planned doses (percentage of gamma‐index values less than unity of 88.3% and standard deviation 9.5%). Upon the introduction of an empirical global small‐field correction factor, the agreement improved greatly (percentage of gamma‐index values less than unity of 94.4% and standard deviation 5.2%) Verification time per plan is approximately 1 minute for the first fraction and approximately 5 seconds for each fraction thereafter.

### Conclusion

Single integrated EPI *in vivo* dosimetry has been successfully extended to VMAT treatments, albeit with the absence of true dosimetric information in the cross‐plane direction. We conclude that 1D dosimetric verification using single EPIs can supplement, but not replace, pretreatment patient specific dosimetric QA.

**MO‐B‐Salon EF‐02**

## Predicting Treatment Couch Coordinates to Reduce First Fraction overrides

S Lacey^*^ J Antolak

*Mayo Clinic, Rochester, MN*

### Purpose

To develop a tool for predicting treatment couch coordinates based on CT simulation data and demonstrate its effectiveness at reducing first fraction couch coordinate overrides.

### Methods

A spreadsheet was developed to calculate treatment couch coordinates based on measurements of two diverging wires embedded in the couch top of the CT simulator. One wire is parallel to the longitudinal axis of the couch, whereas the second wire remains parallel to the couch surface but is angled approximately 5° to the longitudinal axis. Lateral and vertical coordinates are determined from the differences in X and Y coordinates between isocenter and the first wire. The lateral distance between the two wires measured in the axial plane containing isocenter is used to determine the longitudinal coordinate. The calculations assume standardized indexing of immobilization at simulation and treatment. Calculated couch coordinates were compared to actual treatment coordinates recorded for 140 patients. The number of first fraction couch overrides was counted and compared to the number that would have occurred if default couch coordinate values had been used.

### Results

For the 153 unique isocenters that were treated, only 12 (8%) required a couch override for the first fraction. In comparison, if default couch coordinates were used, then 150 (98%) would have a couch override for the first fraction. Of the 12 overrides, seven were expected due to an intentional change in indexing.

### Conclusion

The described technique for calculating treatment couch coordinates has been implemented for nearly all patients at multiple radiotherapy centers. A significant reduction in first fraction couch coordinate overrides has been demonstrated. In addition to adding a quality control check at the first treatment, the elimination of the routine first fraction couch override is expected to increase patient safety by combating override desensitization.

**MO‐B‐Salon EF‐03**

## 3‐D Reconstruction of Radiation isocenter for Stereotactic Radiosurgery

M Carlson,^1*^ G Luo,^2^ A Nelson,^1^ G Ding^1^

*Vanderbilt University,^1^ Nashville, TN, Vanderbilt University Medical Ctr,^2^ Nashville, TN*

### Purpose

To develop software that performs quantitative analysis from portal image data of the radiation isocenter shape and position in space, relative to the linac in‐room laser, for SRS treatments.

### Methods

A suitable phantom BB (i.e., Winston‐Lutz (W‐L) phantom) was aligned to laser isocenter while a radiosurgery cone is centered on the gantry crosshairs. The largest cone available (15 mm) was used to produce a suitable outline around the phantom. Portal images were created in cine mode during a 360° arc. The developed software analyzes each phantom image displacement, then creates a 3D back‐projected volume representing the average isofluence from a complete arc. The surface demarcated by 80% fluence intensity is used to represent mechanical/radiation isocenter. The program output includes shifts required to bring the mechanical and laser isocenters into coincidence. Tests were conducted to assess sensitivity, accuracy, and reproducibility of the method.

### Results

The projected portal image pixel size at isocenter for 140 cm SID is 0.5 mm. The algorithm uses 50% intensity averages to find the cone and phantom centers to a measured precision of 43 microns for a static setup. This far exceeds measured radial magnitude of cone‐mount setup error which was found to be 0.37 mm±0.14 mm. The software, from reconstruction of isocenter, was able to detect intentional phantom displacements (3.0 mm, 2.0 mm, and 1.0 mm in the coronal, transverse, and sagittal dimensions, respectively) to within a 0.3 mm range of visual detectability.

### Conclusion

The software analysis provides both more accurate quantitative data than the traditional W‐L test as well as a 3D rendering of average radiation isocenter over an entire arc. As a result, it can quantitatively assess the degree of gantry mechanical displacement using the fluence back‐projection, and generate laser shifts which allow for easy and accurate readjustment of linac room lasers.

**MO‐B‐Salon EF‐04**

## Investigation of x‐Ray Attenuation Properties of organs Within the Body and Head of Cadaveric Subjects Vs. living Patients for the Validation of CT organ Dose Measurements

A Mench^1*^ L Sinclair^1^ T Griglock^2^ B Cormack^1^ J Sirera^1^ M Arreola^1^

*University of Florida,^1^ Gainesville, FL, Oregon Health and Science University,^2^ Portland, OR*

### Purpose

To investigate how postmortem changes observed in human tissue affect X‐ray attenuation characteristics and confirm the validity of using cadaveric subjects for direct organ dose studies in computed tomography (CT).

### Methods

In order to assess the effects of postmortem changes on the X‐ray attenuation properties of human tissue, and consequently on absorbed dose, seven cadaveric subjects and five living patients spanning a range of body mass indices (BMIs) from 17 to 49 were analyzed in this study. Head‐and‐body CT scans were used to delineate regions of interest (ROIs) encompassing various organs and to obtain average Hounsfield units (HUs) for each region. The calculated effective X‐ray energy was used to determine attenuation coefficients based on these HUs and to draw comparisons between those from organs of cadavers and living patients.

### Results

Most organs showed attenuation coefficients differing by less than 1% between cadaveric subjects and living patients. Breast tissue differed by 2.02% and lungs without the presence of fluid differed from living patients by 2.77%. More significant differences in attenuation were found where fluid had infiltrated the cadaveric lungs and caused a discrepancy of 10.54%. The use of cadavers with minimally fluid‐filled lungs is still clinically relevant as they represent patients with pleural effusions or pneumonia. The results remained consistent with those found in other literature.

### Conclusion

The findings show excellent agreement in attenuation properties between the organs of cadaveric subjects and those of live patients, with the exception of lungs with high levels of fluid retention. This confirms the accuracy of using cadavers for measurement of average organ doses delivered to patients during routing CT examinations.

**MO‐B‐Salon EF‐05**

## Clinical Dose Response Characterization of the PTW oCTAViUS Detector 1000 SRS

J Driewer^*^ B Morgan, D Zheng, S Zhou

*University of Nebraska Medical Center, Omaha, NE*

### Purpose

The PTW OCTAVIUS 1000 SRS is a novel planar array of 977 liquid ionization chambers on fine grid spacing. In this study, we examine the dose response of the 1000 SRS and demonstrate its use for 2D IMRS QA.

### Methods

The 1000 SRS (PTW, Germany) was exposed by a Novalis 6 MV beam (BrainLAB AG, Feldkirchen, Germany) for dosimetric tests. Response was characterized by: reproducibility, linearity, nominal dose rate, field size and depth, angular dependence, and TMR measurements. Finally, a 15 beam IMRS plan was computed and delivered on a 1000 SRS phantom.

### Results

A reproducible and linear dose response over 5 cGy to 6 Gy was observed. Response per cGy was within 0.2%. A dose‐rate dependence of nearly 3.5% from 160 MU/min to 800 MU/min was seen, whether the device was cross‐calibrated at 160 or 480 MU/min. Response dependence on field size and depth was observed and smallest at shallow depths. The angular dependence was about 2% when the beam entered from the back of the detector. and was asymmetric between left and right side irradiations. Gamma analysis on measured and planned IMRS dose distributions yielded 95.6% passing rate at 2%/2 mm and 99% at 3%/3 mm, which compared favorably with standard film and ionization chamber measurements.

### Conclusion

A linear, reproducible dose response over a wide dose range indicates that the detector would be suitable for varying dose/segment and dose levels often seen in IMRS patient specific QA. Dense detector packing contributed to acceptable QA results on a small IMRS field. A nearly 3.5% difference over a range of nominal dose rates, as well as field size and depth dependence, indicates that the choice of cross‐calibration field size and dose rate should be driven by clinical practice. Angular dependence may be mitigated by orienting the detector perpendicular to the beam central axis.

Research supported in part by an equipment grant from PTW‐USA.

**MO‐B‐Salon EF‐06**

## Variation of imaging Dose and image Quality with Tube Potential in Body Perfusion CT. An Analysis Using TG111 Formalism

M Axente,^*^ D Hristov

*Stanford University Cancer Center, Palo Alto, CA*

### Purpose

To investigate the variation of imaging dose and image quality with tube potential in body perfusion protocols using the task group 111 dosimetric formalism.

### Methods

TG111 recommendations were followed in choosing the phantom, dosimetric equipment, and methodology. Specifically, equilibrium doses were measured centrally and peripherally, and an average planar equilibrium dose was determined for each tube potential, for a reference set of exposure parameters (collimation, pitch, filtration) on a Siemens Somatom Definition AS+ scanner (Siemens Medical Solutions, Malvern, PA). This was compared to an equivalent dosimetric index, the weighted CT dose index. To analyze the image quality variation with tube voltage, inserts with different concentrations of iodinated contrast agent were placed in the phantom. The imaging was done using a 144 mm, 1.5 sec body perfusion protocol in shuttle mode, while the imaging doses were kept constant.

### Results

Equilibrium dose increases with tube potential following an approximate kVp cube function. Furthermore, there is a significant underestimation of the imaging dose as reported based on CTDI measurements, ranging from 51% at 70 kVp to 58% at 140 kVp. CNR values generally decrease with increasing tube potential. Acceptable contrast‐to‐noise ratios values (70 kVp: 1.7; 80 kVp: 1.3) for the lowest concentrations of agent used, indicating that lower patient doses could be utilized for imaging.

### Conclusion

Significant dose underestimation by the console report was noted. When imaging different concentrations of imaging agents, it was observed that for the same imaging dose lower kVp values, and inherently lower imaging doses can produce acceptable CNR images for all investigated concentrations. Future work will include further investigations in the TG111 parameterized dose metrics and assumptions. A full dosimetric evaluation of current body perfusion protocols will determine the exposure parameters associated with their clinical implementation. Furthermore, the scanning speed, sampling frequency and tube current limits will also be investigated.

Research funded by Siemens Medical Solutions.

**MO‐B‐Salon EF‐07**

## Volume‐based evaluation of Geometric Distortion in Magnetic Resonance imaging Using 3D Template Matching

K Huang^1*^ Y Cao^1^ U Baharom^2^ J Balter^1^

*University of Michigan,^1^ Ann Arbor, MI, Integrated Medical Technologies,^2^ Troy, NY*

### Purpose

Treatment planning with solely MR images is drawing increased interest in radiation therapy. One of the major issues potentially limiting such use is the possibility of geometric distortion inherent in MR images. Previously the evaluation of MR distortion has been either done in 2D or in 3D using 2D images in AP, SI, and LR planes. We proposed a method to determine the distortion of MR image volume in a real 3D approach.

### Methods

A large phantom with 3D array of spheres filled with contrast was scanned with a 3T MRI simulator (Siemens Skyra, Siemens Medical Solutions, Malvern, PA) using T1 3D‐Vibe protocol (TR 4.39 ms and TE 2.03 ms). 3D template matching using templates of sphere and half spheres with different orientations were used to locate the sphere centers. The normalized cross‐correlation (NCC) value measures how well the image pattern is matched with templates. The half‐sphere templates are used when the NCC value is below threshold. To remove the shift of the sphere centers induced by the physical rotation and translation of the phantom, the Procrustes method was applied. Then the distortion at each sphere center is calculated and the distortion map of the whole image space was generated by interpolation.

### Results

Among the half‐sphere templates used in matching, down half‐sphere was most frequently used (83%), followed by left/right half‐sphere (8% each). Among all the sphere centers, 80% have distortion less than 1 mm, 14% have 1 to approx. 1.5 mm distortion, 4.4% have 1.5 to approx. 2 mm distortion, and 1.6% have distortion more than 2 mm. The coordinates of the location closest to center with >2 mm distortion is at (11.0,−17.8,−4.7).

### Conclusions

This work successfully demonstrated 3D template matching is effective to locate the coordinates of a sphere center in 3D simultaneously. With this method, a high‐fidelity MR distortion map was able to be generated in less than 1 minute.

**MO‐B‐Salon EF‐08**

## Direct organ Dose Measurement for Multidetector Computed Tomography Utilizing Cadaveric Subjects

L Sinclair^1*^ A Mench^1^ T Griglock^2^ B Cormack^1^ S Bidari^1^ M Arreola^1^

*University of Florida,^1^ Gainesville, FL, Oregon Health and Science University,^2^ Portland, OR*

### Purpose

While CT has allowed for great advances in the field of diagnostic imaging, this progression has not ensued without careful attention to the radiation dose associated with its use. There remains a critical need to accurately assess organ doses resulting from these procedures. This research aims to directly measure organ doses by utilizing cadaveric subjects.

### Methods

Techniques in a CT exam are tailored according to patient size and scanner‐specific parameters. To account for this, four cadaveric subjects of body mass indices (BMI) ranging from 17 to 49 (BMI) were utilized for direct organ dose measurement on a 320 slice CT scanner. A dosimeter placement system was previously developed which utilizes optically stimulated luminescent dosimeters (OSLDs). The OSLDs were placed on the skin and within the following organs: thyroid, lungs, breasts, liver, stomach, small intestine, large intestine, uterus, and ovaries. The location and number of dosimeters were based on the size and distribution of each organ, with a maximum of 48 dosimeters used for each scan. A variety of clinically accepted CT protocols was examined, including chest (C), abdomen (A), pelvis (P), CAP, 3‐phase liver, pulmonary embolism, and trauma protocols.

### Results

Average and maximum organ doses were recorded for each exam. For a CAP exam, average organ doses for all subjects ranged from 8–26 mGy for organs in the primary beam. The highest organ doses were observed in the two subjects that classified as obese, with BMIs over 30.

### Conclusion

Direct organ dose measurement resulting from CT exams is possible and has been completed for a range of representative adult patient sizes and clinical protocols with this research. This methodology can be extended to any CT scanner, protocol, or patient size, resulting in more accurate dose data. Additionally, these directly measured organ doses can be compared to estimated values obtained from other methodologies.

**MO‐B‐Salon EF‐09**

## Analysis of Automatic Match Results for CBCT localization of Conventionally Fractionated lung Tumors

M Grams^*^ L Brown, D Brinkmann, D Pafundi, D Mundy, S Park, Y Garces, K Olivier, L Fong de los Santos

*Mayo Clinic, Rochester, MN*

### Purpose

To evaluate the dependence of an automatic match process on the size of the user‐defined region of interest (ROI), the structure volume of interest (VOI), and changes in tumor volume when using CBCT for lung tumor localization, and to compare these results to a gold standard defined by a physician's manual match.

### Methods

Daily CBCT images for 11 lung cancer patients (109 fractions) treated with conventionally fractionated radiotherapy were retrospectively matched to a reference CT using Varian's OBI software (Varian Medical Systems, Palo Alto, CA) and a three‐step automatic matching protocol. Matches were performed with three ROI sizes (small, medium, large), both with and without a structure VOI (ITV or PTV) used in the last step. Additionally, matches using an intensity range isolating the bony anatomy of the spine were performed. All automatic matches were compared with a physician's manual match.

### Results

Automatic match results depend on ROI size and the structure VOI. Compared to the physician's manual match, automatic matches using the PTV as the structure VOI and a small ROI resulted in differences greater than or equal to 5 mm in only 1.8% of comparisons. Automatic matches with a large ROI and no VOI resulted in differences of at least 5 mm in 30.3% of comparisons. Automatic matches to the spine resulted in differences of at least 5 mm in 21.1% of comparisons. Differences between manual and automatic matches using the ITV as the structure VOI increased as tumor size decreased.

### Conclusions

This study illustrates the effectiveness of an automatic matching protocol for lung cancer patients even when large changes in tumor volume occur. Optimal matching parameters included using a small ROI and the PTV as the structure VOI on the last step of the automatic match process. Users of automatic matching techniques should carefully consider how user‐defined parameters affect patient localization.

**MO‐B‐Salon EF‐10**

## Effect of Human Perception on Winston‐lutz QA Process

A Moghadam^*^ R Hamilton, C Watchman

*University of Arizona, Tucson, AZ*

### Purpose

This study demonstrates the effect of human bias in performing the QA tests and specifically quantifies the amount of uncertainty due to observer bias for the case of Winston‐Lutz (WL) test for stereotactic radiosurgery (SRS).

### Methods

24 WL tests for previously treated patients were used in this analysis. Each test was analyzed using a MATLAB program (The MathWorks, Natick, MA) which required the user to place a series of circles around the BB and cone regions. The positional differences were then determined by the program. A second program was developed to analyze the displacement of the circle centers automatically. The automated program uses center of mass calculation along with an optimization algorithm to find the outer irradiation circle and BB shadow circle. The optimization process involves finding the circle which maximizes the summation of pixel values enclosed by the circle for the outer irradiation circle. The BB circle is found by a minimum summation of pixel values. The difference in the measured deviation between the two methods of measurement was then evaluated.

### Results

The average displacement measured by the manual process was found to be always smaller than the amount measured by the automated program. Our analysis also showed that 17% of the cases examined would have failed the WL criterion had the automated method been used.

### Conclusion

Observer bias in WL analysis is a significant factor in the QA process. This analysis indicated that nearly 1/6 of observer determined cases would fail as compared to the objective method. Also, it appears that the observer will innately conclude the deviation to be smaller than the automated method describe.

**MO‐B‐Salon EF‐11**

## evaluation of GAFCHRoMiC xRQA2 Film for Varian oBi KV Scanner Dose Measurements

A Turner^*^ J Little

*University of Arizona, Tucson, AZ*

### Purpose

To assess the magnitude of energy dependence of GAFCHROMIC XRQA2 for dose measurements performed using the Varian On‐Board Imager (OBI) kV imaging system.

### Methods

All measurements were obtained with the OBI (Varian Medical Systems, Palo Alto, CA) kV source at 120 kVp with a source to isocenter distance of 100 cm, 50 cm squared field, and the full bowtie filter. Reference measurements were obtained in air with an approximately energy‐independent CC13 ionization chamber. Exposure profiles were measured across the bowtie filter over the entire isocenter plane, with increments of 2.5 to 5 cm, for longitudinal positions ranging from negative 20 cm to positive 20 cm. Film was exposed with a source to film distance of 40.6 cm, digitized, and projected to the isocenter plane. Film measurements were corrected for background and noise and the red channel was extracted. Both film and ionization chamber measurements were normalized to the respective isocenter value. The normalized film and exposure profiles were compared for each longitudinal position measured with the ionization chamber.

### Results

Examination of the normalized profiles indicates that there are considerable differences between the film and ionization chamber measurements across the bowtie filter plane regardless of longitudinal position. The average normalized difference across all measurements was 33.4%±13.6%. In general, differences increased with radial distance from the isocenter until approximately 7.5 to 10 cm and then gradually decreased.

### Conclusion

Since ionization chambers are approximately energy independent, the differences illustrate that GAFCHROMIC XRQA2 film may exhibit substantial energy dependence for the range of beam qualities across the isocenter plane for Varian OBI kV scanners. The variation in beam qualities is due primarily to the varying thickness of the bowtie filter, resulting in varying levels of beam hardening. It may be possible to correct film measurements for controlled measurement setups. Future work will be done to evaluate different tube voltage levels, bowtie filter settings, and film processing protocols.

**MO‐B‐Salon EF‐12**

## Optimization Strategies for Midline and Peripheral Tumours for iMRT and RapidArc ‐ A Phantom Study

S Kumar^*^ A Silpa, B Resmi, C Suja

*Malabar Cancer Centre, Kannur, Kerala, India*

### Purpose

To evaluate the optimization strategies for midline and peripheral tumors for IMRT and RapidArc treatments.

### Methods

Homogeneous phantom was CT scanned and PTV was delineated for two different positions (midline and periphery). Two organs at risk with different shapes (organ at risk 1, organ at risk 2) were created. Different plannings were done with organ at risk 1, placed at distance of 0.5 cm and 2 cm. Also organ at risk 2, placed at a distance of 1 cm and 2.5 cm from the border of PTV along the central axis. Planning has been done for IMRT using nine fields and RapidArc (Varian Medical Systems, Palo Alto, CA) with double arc. Beam has been equally placed for IMRT plans and RapidArc plans utilize full 360° gantry rotation.

### Results

Dose homogeneity was almost similar for tumors in the midline where organs at risk are far. But RapidArc plans show superior dose homogeneity in PTV, when the target is situated at the periphery and organs at risk are very near (HI −2.67 for Rapid Arc and HI 4.03 for IMRT). Target coverage was better for all RapidArc plans with maximum CI 1.01. The sparing of organ at risk in terms of the maximum dose was better in RapidArc. A considerable reduction in organ at risk mean dose (12.37% for organ at risk 1 and 10.23% for organ at risk 2) was observed with RapidArc technique for peripheral tumors. For healthy tissue, no significant changes were observed in terms of the mean dose and integral dose. But RapidArc plans show a reduction in the volume of the healthy tissue irradiation above V10 Gy for targets at the periphery and OAR near.

### Conclusion

Either IMRT or RapidArc can be chosen for tumors in the midline. In particular, RapidArc treatment can be recommended for tumors which are situated at the periphery.

**DIAGNOSTIC SYMPOSIUM ‐ SAM**

**SALON BCD**

***ADVANCED FLUOROSCOPY: CARDIAC CATH LAB / DOSE TRACKING (FLUORO AND CT)***

**MO‐B‐Salon BCD‐01**

## X‐Ray Fluoroscopy imaging in the invasive Cardiac laboratory: Medical Physics Support of a Contemporary Practice

K Fetterly

*Mayo Clinic, Rochester, MN*

While there are several imaging technologies that are used to diagnose and treat cardiovascular disease, X‐ray fluoroscopy remains the primary modality used for guidance during invasive cardiovascular procedures. From the clinical perspective, fluoroscopy system quality is a combination of image quality and patient radiation dose. Image resolution, contrast, and noise are highly variable between systems and operational modes. Patient radiation skin dose is a principle safety concern and is highly dependent on the patient, clinical procedure, and imaging system design and operation. The purpose of this presentation is to provide information relevant to testing and optimizing interventional X‐ray fluoroscopy systems to support a wide range of clinical imaging tasks. The various imaging technologies routinely used in a contemporary invasive cardiology laboratory will be introduced. Comprehensive methods to assess quality and clinically relevant skin entrance air kerma from interventional fluoroscopy systems will be presented. The relevance of image quality metrics will be presented in the context of the various clinical imaging tasks performed. System optimization with respect to patient dose requires a desire to reduce dose while not affecting the clinical utility of the images. This strategy provides the potential for substantial patient dose reduction. Modern X‐ray system design and control offer many possibilities for radiation dose reduction. Specific methods to minimize patient radiation dose while not adversely affecting patient care will be presented.

### Learning objectives

1. Understand the need for and methods used to fully characterize interventional fluoroscopy system performance as it relates to the clinical imaging task.
2. Recognize system technical parameters that can be adjusted to reduce patient radiation dose.
3. Appreciate the potential for active management to reduce patient radiation dose without adversely affecting the clinical utility of the images.

**MO‐B‐Salon BCD‐02**

## Radiation Dose informatics: Using the Tools of Six Sigma to improve Radiation exposure Prescription

W Pavlicek

*Mayo Clinic Arizona, Scottsdale, AZ*

New commercial software products can capture patient exposure information, store these data and web‐enabled reports to provide users with radiation prescriptions from diagnostic X‐ray exams, including CT and Fluoroscopy. These tools provide highly relevant patient‐specific and summary information that is enormously useful and actionable for ensuring that the intended exam exposure took place, in fact. Radiation dose to organs can be computed for CT exams and skin dose can be computed for fluoroscopy use. Excursions from intended prescriptions can be easily identified for a facility, device, protocol or patient. Six Sigma (SS) is well suited as the contextual approach of using these software tools as the improvements in accuracy and optimal prescription of X‐rays for diagnosis deal with the unwanted variability that a ‘process'‐driven service inherently generates. Basic SS concepts introduced in this talk derive from DMAIC (De‐fine, Measure, Analyze, Improve, and Control), the organized approach for systems engineered changes having improvements in quality as the goal. Data show that prescriptions of X‐ray as currently configured with these devices allow for wide ranges of patient exposures (machine outputs). At its basis, the range of patient size, geometry of patient positioning, and the image quality needs of clinically needed but widely ranging types of diagnostic exams or therapeutic interventions with fluoroscopic and CT. Included in the measures of observed variability is the complexity of operator selectable device controls. This increases the opportunity for nonuniform training of staff leading to further variations from the ideal. Six Sigma recognizes that the primary need is to reduce variability from the intended dose prescription goal. It embraces team involvement, continuous improvement, standardization, and education as basic to improving outcomes. DMAIC notes that it is not the individual but the process which is faulty. Specific examples of data gathered by these software tools will be used to show how one can monitor, understand, and facilitate improvements for CT and fluoroscopy.

### Learning objectives

1. To review the key concepts of Six Sigma as a tool for the strategic approach of refining X‐ray exposure prescriptions with CT and fluoroscopy.
2. To demonstrate several powerful, newly available, and important software tools that allow key Six Sigma concepts to be addressed, including the ability to provide:
  - Measures of variability in a process, not as a human fault
  - Quantified measures of quality prescription
  - Records of patient data that are timely and thus actionable
  - User‐specific behavior training tools
3. To give specific examples of the use of informatics in reducing variability of X‐ray exposure prescription, including:
  - Patient centering/CT radiographs
  - Technologist virtual teaching tool for CT
  - Physician virtual teaching tool for fluoroscopy
  - Protocol specific fluoroscopic and CT exposures

**PROFESSIONAL SYMPOSIUM**

**SALON EF**

***MEDICAL PHYSICS PRACTICE GUIDELINES***

**MO‐C‐Salon EF‐02**

## Medical Physics Practice Guidelines

D Cody^1*^ J Fontenot^2*^

*U.T.M.D Anderson Cancer Center,^1^ Houston, TX, Mary Bird Perkins Cancer Center,^2^ Baton Rouge, LA*

Recently, the American Association of Physicists in Medicine (AAPM) established a Medical Physics Practice Guidelines (MPPG) initiative to provide a clear and concise description of the minimum level of medical physics support that the AAPM would consider to be prudent in all clinical practice settings. As accreditation of clinical practices becomes more common, MPPGs will be crucial to ensuring a consistent benchmark for accreditation programs. MPPG reports will be freely available to the general public. Accrediting organizations, regulatory agencies, and legislators will be encouraged to reference these MPPGs when defining their respective requirements. Support includes, but is not limited to, staffing, equipment, machine access, and training. This session will describe the purpose and scope of MPPGs, the procedure for the development of a MPPG, and reports from TG‐226 MPPG Therapy #1 and TG‐225 MPPG Diagnostic #1. TG‐226 MPPG Therapy #1 has completed their report, entitled ‘'Evaluation and quality assurance of x‐ray based image guided radiotherapy system”. Sections of the TG‐226 report include a review of image‐guidance technologies, a description of the treatment team and their responsibilities in IGRT system use and management, and recommendations for minimum commissioning and quality assurance practices. Sample process descriptions, including equipment and methods, for recommended quality assurance practices are provided. Finally, staffing estimates and resource requirements needed for IGRT system management are provided. TG‐225 MPPG Diagnostic #1 has completed their report, entitled ‘'CT Protocol management and review”. Sections of the TG‐225 MPPG Diagnostic #1 report include a description of the CT protocol management and review process, team members and their responsibilities, and a general description of the overall CT protocol management and review process. Issues such as recommended review frequency, acquisition parameters to review, documentation of the review process, and refresher education for the CT protocol management and review team are addressed. This MPPG acknowledges that CT protocol management and review are essential to providing images with sufficient quality and ensuring patient safety. It also acknowledges that this process is very challenging and identifies areas for development, including improved tools from the CT manufacturers that are sorely needed in order to make this process more efficient.

### Learning objectives

1. Understand the concept, scope, and process of MPPG from the AAPM.
2. Understand the goals, methodology, and recommendations of TG‐226 MPPG Therapy #1.
3. Understand the goals, methodology, and recommendations of TG‐225 MPPG Diagnostic #1.

### Speakers

Jonas Fontenot, Chair of TG‐226 MPPG Therapy #1

Dianna Cody, Chair of TG‐225 MPPG Diagnostic #1

**PROFESSIONAL SYMPOSIUM ‐ INTERACTIVE**

**SALON BCD**

***CONTROVERSIAL MEDICAL PHYSICS ISSUES***

**MO‐C‐Salon BCD‐01**

## Controversial Medical Physics issues

J Clements^1*^ P Halvorsen^2*^ K Antes^3*^ D Jordan^4*^

*Texas Health Presbyterian Hospital Dallas,^1^ Dallas, TX, Alliance Imaging/Alliance Oncology,^2^ Newton, MA, Presbyterian Healthcare System,^3^ Dallas, TX, University Hospitals Case Medical Center,^4^ Shaker Heights, OH*

This is a two‐hour panel discussion of controversial and professional topics in medical physics. Specific topics have been identified for debate and audience participation. When applicable, controversial scenarios will be addressed with AAPM publications including task group reports, position statements, and editorials from medical physics journals.

### Learning objectives

1. Discuss the meaning of ethics and professionalism.
2. Review relevant AAPM publications relating to identified controversial topics.
3. Discuss controversial topics and discover audience opinion from audience interaction.

**THERAPY SYMPOSIUM ‐ SAM**

**SALON EF**

***DATA INTEGRITY AND ELECTRONIC CHARTING (EBRT AND BRACHYTHERAPY)***

*MO‐D‐Salon EF‐01*

## Data integrity and electronic Charting (eBRT and Brachytherapy)

L Benedetti (Burgess)^1*^ R Siochi^2*^

*William Beaumont Hospital,^1^ Royal Oak, MI, University of Iowa,^2^ Iowa City, IA*

The Electronic Medical Record (EMR) in Radiation Oncology presents the additional challenge of controlling medical equipment, setting it apart from the rest of the Hospital Information Systems Databases. Errors in these records can have potentially fatal consequences, underscoring the need for careful implementation, testing, and maintenance. An understanding of the information technology infrastructure, data storage, and data flow in radiation oncology is crucial for the design of quality assurance policies and procedures that must be performed to maintain data integrity. Such procedures address the data transfer among various subsystems encountered as the patient moves from the initial consult to treatment. These tests should check that treatment information, not just data, is preserved during a transfer between pairs of subsystems. The relevant principles outlined in the rapid communication and the draft TG201 full report will be reviewed, followed by a discussion of the logistics involved in a successful implementation of the EMR. This success depends on the support of not only radiation oncology personnel but also hospital IT staff. Personnel involved in the information exchanges with the EMR will need to evaluate all current processes and modify them to be compatible with the EMR. The modifications should facilitate a safer and more accurate delivery of radiation, not only for external‐beam radiation therapy (EBRT) but also for brachytherapy. They should also exploit the advantages of an EMR to make processes more efficient and maintain the culture of safety. Implementation of the EMR in brachytherapy requires the collaboration of the Radiation Oncology Department and the hospital Radiation Safety Department to ensure compliance with the NRC or State requirements. An overview of the clinical implementation process of electronic charting for both external beam and brachytherapy will be presented.

### Learning objectives

1. Review basic IT infrastructure concepts that are used in the deployment of Electronic Medical Records in Radiation Oncology.
2. Understand the principles of QA and QC data transfer test design.
3. Discuss department staffing and process evaluation for successful EMR implementation.
4. Understand the workflow for implementation of the EMR for EBRT and Brachytherapy.

**DIAGNOSTIC SYMPOSIUM ‐ SAM**

**SALON BCD**

***RADIATION SAFETY: TIPS FOR THE DX MP / SHIELDING FOR MULTI‐MODALITY TECHNOLOGY***

**MO‐D‐Salon BCD‐01**

## Fifty Shades of Gray: A Medical Physicists Guide as RSo

K Nelson

*Mayo Clinic, Jacksonville, FL*

In a complex medical environment, ensuring compliance with ionizing radiation regulations and interpretations can be challenging, especially if an individual also has additional responsibilities beyond radiation safety. Participants will be expected to have a working knowledge of the applicable ionizing radiation regulations as they will not be reviewed in great detail during this session. Instead, issues identified during agreement state or NRC inspections will be reviewed. Particular focus will be paid to issues involving radioactive materials: RSO training requirements, use of unsealed byproduct material, required records, general technical requirements, security, and a review of NRC enforcement actions.

### Learning objectives

1. Review training and education requirements for a Radiation Safety Officer.
2. Obtain a better understanding of typical regulatory issues encountered in the medical use of byproduct material.
3. Review recent NRC enforcement actions against medical use licensees.

**MO‐D‐Salon BCD‐02**

## Radiation Safety

M Martin

*Therapy Physics, Inc., Gardena, CA*

Combined imaging modalities of PET/CT and PET/MRI are currently available in the United States. Designing the shielding required for these dual modality units is complex and requires knowledge of both modalities for these pieces of equipment. Design considerations for PET/CT scanners include delivery and storage of the radioisotope, dose preparation and administration, uptake rooms and restroom facilities, scanning room, control room, postscan requirements, and accompanying persons. The primary radioisotope used for whole‐body PET scans is Fluorine‐18 FDG while Rb‐128 is used for cardiac studies. Ideally, the department is laid out with the highest level of activity toward an exterior wall and away from the reception and public areas of the department. Individual calculations for each of these areas must be made in accordance with the recommendations of AAPM Task Group 108 Report. Occupancy factors for each area vary and must be taken into consideration. Additional separate calculations must be performed for the expected exposures from CT scanner, particularly if the CT scanner is used independently from PET acquisitions. The same type of considerations must be made for the MRI/PET scanners in that the shielding needed for MRI will not provide protection from the radiation exposure from the CT scanner, while the usual lead shielding used to provide radiation protection for the CT scanner will not prevent artifacts in the MRI suite or excess magnetic fields in the control room area. Radio frequency (RF) shielding works to prevent exterior RFI (Radio Frequency Interference) from negatively affecting the operation of the MRI and the digital image being produced. An RF shield encompasses all six sides of a room. The design and construction challenge with RF enclosures occurs when addressing the need to connect or penetrate certain necessary applications such as the RF door, RF window, and air vents.

### Learning objectives

1. Attendees will be aware of the required radiation shielding needed to limit the radiation exposure from the use of PET isotopes in the Diagnostic Imaging Department.
2. Attendees will be aware of the required radiation shielding needed to limit the radiation exposure from the use of the CT scanner in connection and independent of the PET portion of the PET/CT scanner
3. Attendees will be aware of the required shielding needed for a MRI/PET scanner to prevent artifact generation within the MRI scanner room due to outside interference from RF signals.
4. Attendees will be aware of the required magnetic shielding needed for a MRI/PET scanner to minimize the magnetic exposure levels for staff to established safe levels while allowing entry into the room, observation of the patient, and ventilation for the scanner room.

**TUESDAY, MARCH 19**

**THERAPY SYMPOSIUM ‐ SAM SALON EF**

***CLINICAL TRIALS***

**TU‐A‐Salon EF‐01**

## Clinical Trials

J Moran^1*^ Y Xiao^2*^

*University Michigan Medical Center,^1^ Ann Arbor, MI, Thomas Jefferson University Hospital,^2^ Philadelphia, PA*

Several studies have demonstrated the importance of quality with respect to outcomes in multidisciplinary clinical trials. This session will present on the work of AAPM Task Group 113 with respect to improving consistency in clinical trials. Examples which have demonstrated the importance of consistency in different aspects of the radiation therapy process will be presented. The role of audits, credentialing, and benchmarks will also be discussed, with examples demonstrating important improvements in the quality of clinical trials. Then, a proposed new framework for supporting clinical trials will be described: the Clinical Trial Quality Assurance Imaging and Radiation Oncology Core Group (IROC). An important aspect of the proposed group is to acknowledge the vital role of multimodality imaging in identifying target volumes, treatment assessment, and volumetric image guidance for advanced technology trials. Infrastructure requirements for an efficient workflow will be described along with some examples of how this infrastructure currently works. The infrastructure supports investigators leading the multidisciplinary trials and allows them to provide timely and effective feedback to the investigators at the individual institution who are enrolling patients in clinical trials. Finally, a vision for future quality assurance needs will be presented.

### Learning objectives

1. To report on the additional requirements for multidisciplinary clinical trials.
2. To describe examples demonstrating how audits, credentialing, and benchmarks can support quality in clinical trials.
3. To describe the benefits and requirements of close coordination of RT and imaging QA.
4. To describe current and future needs for an efficient informatics infrastructure for clinical trials.

**DIAGNOSTIC SYMPOSIUM ‐ SAM**

**SALON BCD**

***GENERAL DIAGNOSTIC: DENTAL X‐RAY / PREPARING FOR AN ACR SITE VISIT***

**TU‐A‐Salon BCD‐01**

## General Diagnostics

R Pizzutiello

*Upstate Medical Physics, Victor, NY*

Recent improvements in digital dental imaging have created new opportunities for medical physicists to contribute to its safety and quality. Cone‐beam computed tomography (CBCT) has dramatically grown in to many dental specialty practices, and even into the offices of general dentists. In 2009, NCRP Report #160 estimated the order of magnitude of dental X‐ray images to 100 million dental X‐ray images are acquired annually in the USA. As digital dental imaging technology has advanced, increasingly “user friendly” systems have created the opportunity for suboptimally trained personnel to deliver more radiation than is needed for digital dental exams (think “Dose Creep”, and more). Recent reports on dental imaging, including CBCT, from the American Dental Association, FDA, and IAEA will be summarized. This presentation will begin with common problems encountered in film‐based dental imaging, but will primarily be focused on the digital dental imaging environment. Methods for evaluating the performance of dental radiography, cephalometric, panoramic, and CBCT will be presented. The medical physics requirements for the Intersocietal Accreditation Commission CT Accreditation Program for dental CBCT systems will be reviewed.

### Learning objectives

1. Understand the impact of digital imaging on the dental radiation safety.
2. Understand the methodology for performance evaluations of dental imaging systems.
3. Understand the medical physics requirements for IAC CT accreditation of dental CBCT systems.

**TU‐A‐Salon BCD‐02**

## The American College of Radiology Random on Site Survey

W Geiser

*UT MD Anderson Cancer Center, Houston, TX*

As part of its accreditation programs, the American College of Radiology (ACR) reserves the right to perform on‐site surveys of any facility at any time during the accreditation process. For mammography, the ACR is actually directed by MQSA to perform on‐site surveys of at least 5% of the facilities it accredits each year. The ACR calls these surveys a Random On‐Site Survey or ROSS. The ACR sends a three‐person team to a randomly chosen facility for the purpose of inspecting that facility to ensure that it is meeting the quality standards set forth in the accreditation program. The team consists of three individuals, a radiologist, a physicist, and an ACR staff member from the appropriate accreditation program. Once the survey is complete, the ACR team performs an exit interview with the facility program directors, to inform the facility of the outcome of the survey. This lecture will give the medical physicist an understanding of the process that the ACR uses to perform a ROSS, the responsibilities of each of the ROSS team members, and the roll the facility medical physicist plays in the inspection process. With this information, the medical physicists will be able to help their accredited facilities come through the inspection with high marks from the survey team.

### Learning objectives

1. Understand the survey process.
2. Understand what documentation is needed at the facility.
3. Be able to help the facility prepare for a random on‐site survey by the ACR

**PROFESSIONAL SYMPOSIUM**

**SALON EF**

***EFFECTIVE COMMUNICATION SKILLS WITH STAFF AND PATIENTS***

**TU‐B‐Salon EF‐01**

## Effective Communication Skills with Staff and Patients

K Levine

*University of Tennessee, Knoxville, TN*

Efficient communication between and among the members of a health‐care team is vital to the care of the patient, and effective communication between members of the health‐care team and the patient is essential for quality diagnosis, in‐ and posthospital care. A breakdown in the communication process at any point may result in an increase in errors or omissions related to patient care and a decrease in patient satisfaction. This presentation will examine three specific communication issues facing health‐care professionals. All members of a health‐care team need to understand that they are communicating to two separate and distinct audiences: (1) members of the health‐care team, and (2) the patients. The members of the health‐care team are well‐trained professionals with a jargon and urgency of their own. Effective communication within this group is based on disseminating information efficiently. Patients often do not understand the jargon used by the medical staff, and they often have many questions. When communicating with nonmedical audiences, the medical staff's primary goal must be to maximize understanding, thus effectiveness over efficiency. This presentation will discuss this tension between effective communication and efficient communication within different medical contexts. A crucial context within the teaching hospital setting is the “hand‐off” meeting. The hand‐off meeting is a daily event between and among interns, residents, and attending physicians that occurs at the end and beginning of a working shift. At this time, information is transferred from one set of medical professionals to another. This presentation will report the findings of an exploratory study into the hand‐off process. The research included the use of naturalistic observations at the shift‐change meetings and of “work rounds” in a Critical Care Intensive Care unit and follow‐up interviews with interns, residents, and attending physicians. The findings suggest that there is a difference between the messages that are sent and the information that the medical professionals would like to receive. Any conflict within a medical team or unit is likely to impact patient satisfaction. This presentation will discuss some early findings of an on‐going study examining conflict within a medical unit to determine whether internal conflict has an impact on the patients and their perceptions of care. Lastly, there are important issues facing the medical profession due to the generation gap between older and younger members of a health‐care team. While not unique to the health‐care field, intergenerational communication issues may impact patient care and doctor–patient communication, as the different age groups have very different expectations regarding effective and efficient communication. Additionally, the impact of the new 80‐hour work week on the training of residents and interns is causing an us‐versus‐them mentality among members of the health‐care team. This presentation will discuss these intergenerational issues by concentrating on the communication‐related issues resulting from the new limits on medical training. The presentation will conclude with suggestions for training techniques, and for future research into effective and efficient communication among medical personnel and between medical staff and patients.

**TU‐B‐Salon EF‐02**

## Effective Communication Skills with Staff and Patients

N Anderson

*Robert Boissoneault Oncology Institute, Ocala, FL*

As a physician for over 32 years, I have grown both in knowledge of medicine and understanding of human nature. The training of a physician can create a gamut from compassionate healers to self‐proclaimed god‐like figures whose “fame” is elucidated for your benefit at the drop of a hat. The latter group of medical doctors have unlimited ability. If in doubt, ask them, and they will provide you detailed justification for their arrogance. The mental framework of a physician — Control vs. Communication, Dictate vs. Discuss, Ego vs. Equal, Superiority vs. Sensitivity, Tyrant vs. Team, Fiefdom vs. Fraternity, Part vs. Patient, Number vs. Name. On the other side of the coin is the patient. In most cases, individuals feel in control when their environment allows the use of familiar skills. Due to a medical situation, control is now lost. One becomes overwhelmed with the totality of their illness or that of a close friend/relative. The “unknown” that the future holds causes one to focus on every word, comment, and statement that is uttered by a professional, or nonprofessional. After all, cancer is the big unknown, and we use an invisible unknown to cure. Such a combination does not comfort one's soul. Remember, even a physician becomes totally dependent on those providing care. All face the same feeling of hopelessness, as the initials behind a name fail to calm emotion. The mental framework of a patient — Secure vs. Scared, Calm vs. Confused, Knowledge vs. None, Reaching for Hope vs. Reaching for Hope while Waiting for the Touch of Compassion/Listening for the Voice of Comfort/Searching for the Answers Between. There are a myriad of staff whose experience with such diverse personalities varies dramatically. Some have the ability to quell the fire of anxiety. Others seem to incite, as gasoline is thrown on the fames. And there you are. You are a medically trained professional attempting to bridge the gap, demonstrating your ability to a physician who may view you as equal colleague or custodian; or to the patient who misspells your professional title, much less understands the vital knowledge you provide for their health. In reality, the goal of medical treatment is not accomplished by one individual. And verbal communication, as you will see, is only one of the tools. This presentation will facilitate your efforts to reach that goal. Over time, the physicians will appreciate your participation in the healing process, as the team provides the patient with an answer to the mystery of medicine. I guarantee the satisfaction you experience will rival your professional achievements. I know this to be the case because I know how the arrogant doctor's brain works. You see … I once was one.
